# Essential Role of σ Factor RpoF in Flagellar Biosynthesis and Flagella-Mediated Motility of *Acidithiobacillus caldus*

**DOI:** 10.3389/fmicb.2019.01130

**Published:** 2019-05-24

**Authors:** Chun-Long Yang, Xian-Ke Chen, Rui Wang, Jian-Qiang Lin, Xiang-Mei Liu, Xin Pang, Cheng-Jia Zhang, Jian-Qun Lin, Lin-Xu Chen

**Affiliations:** State Key Laboratory of Microbial Technology, Shandong University, Qingdao, China

**Keywords:** RpoF, *Acidithiobacillus caldus*, flagellar gene cluster, flagellar synthesis regulation, swarming, Sox system

## Abstract

*Acidithiobacillaceae*, an important family of acidophilic and chemoautotrophic sulfur or iron oxidizers, participate in geobiochemical circulation of the elements and drive the release of heavy metals in mining associated habitats. Because of their environmental adaptability and energy metabolic systems, *Acidithiobacillus* spp. have become the dominant bacteria used in bioleaching for heavy metal recovery. Flagella-driven motility is associated with bacterial chemotaxis and bacterial responses to environmental stimuli. However, little is known about how the flagellum of *Acidithiobacillus* spp. is regulated and how the flagellum affects the growth of these chemoautotrophic bacteria. In this study, we analyzed the flagellar gene clusters in *Acidithiobacillus* strains and uncovered the close relationship between flagella and the sulfur-oxidizing systems (Sox system). The σ^28^ gene (*rpoF*) knockout and overexpression strains of *Acidithiobacillus caldus* were constructed. Scanning electron microscopy shows that *A. caldus* Δ*rpoF* cells lacked flagella, indicating the essential role of RpoF in regulating flagella synthesis in these chemoautotrophic bacteria. Motility analysis suggests that the deletion of *rpoF* resulted in the reduction of swarming capability, while this capability was enhanced in the *rpoF* overexpression strain. Both static cultivation and low concentration of energy substrates (elemental sulfur or tetrathionate) led to weak growth of *A. caldus* Δ*rpoF* cells. The deletion of *rpoF* promoted bacterial attachment to the surface of elemental sulfur in static cultivation. The absence of RpoF caused an obvious change in transcription profile, including genes in flagellar cluster and those involved in biofilm formation. These results provide an understanding on the regulation of flagellar hierarchy and the flagellar function in these sulfur or iron oxidizers.

## Introduction

*Acidithiobacillus* spp. are acidophilic bacteria and widely used in the bioleaching industry. *Acidithiobacillus* species formerly belonged to the genus “*Thiobacillus*" and have been reclassified into the new proteobacterial class *Acidithiobacillia* (Kelly and Wood, 2000; Hudson et al., 2014). All members in *Acidithiobacillus* have remarkable capability of oxidizing various reduced inorganic sulfur compounds (RISCs) to obtain electrons for autotrophic growth, and some of them also have ferrous iron oxidation ability (Harrison, 1984). The isolated bacteria in *Acidithiobacillus* have been classified into seven species: *Acidithiobacillus thiooxidans* (*A. thiooxidans*), *Acidithiobacillus ferrooxidans* (*A. ferrooxidans*), *Acidithiobacillus caldus* (*A. caldus*), *Acidithiobacillus albertensis* (*A. albertensis*), *Acidithiobacillus ferridurans* (*A. ferridurans*), *Acidithiobacillus ferriphilus* (*A. ferriphilus*), and *Acidithiobacillus ferrivorans* (*A. ferrivorans*) (Waksman and Joffe, 1922; Temple and Colmer, 1951; Bryant et al., 1983; Hallberg and Lindstrom, 1994; Falagan and Johnson, 2016; Nunez et al., 2017). *Acidithiobacillus* strains widely exist in iron/sulfur-containing habitats on land or in the sea (Sharmin et al., 2016; Nunez et al., 2017; Kamimura et al., 2018), such as soil (Waksman and Joffe, 1922; Deb et al., 2004), sediments (Nealson, 1997; Otte et al., 2018), hot springs (Arce et al., 2019), iron-sulfur mineral deposits (Johnson et al., 2001; Rowe et al., 2007; Song et al., 2011) and acid mine drainage (AMD) (Colmer and Hinkle, 1947). Due to the unique characteristics in energy metabolism and environmental adaptation, *Acidithiobacillus* strains have been widely used in biohydrometallurgy for non-ferrous metals production (copper, gold, zinc, etc.) (Rawlings, 2005; Valdés et al., 2008a; Donati et al., 2016; Pollmann et al., 2018).

Flagella are important cell appendages for bacterial motility and biofilm formation (Pratt and Kolter, 1998; Lemon et al., 2007; Friedlander et al., 2013). In biomining, the attachment of bacterial cells on mineral surface is a prerequisite for the acquisition of energy-substrates, which also contributes significantly to mineral dissolution process (Africa et al., 2013). The biofilm formation of *Acidithiobacillus* spp. promotes cell attachment and facilitates the oxidization of sulfur or iron deposited in ores (Harneit et al., 2006; Lara et al., 2013; Bellenberg et al., 2014), resulting in the extremely low pH, high concentration of heavy metals and temperature change in the habitat. The flagella-mediated motility and chemotaxis are vital for cells to respond to environmental stimuli (pH, temperature, osmolarity, etc.) and to find nutrients for growth (e.g., energy substrates and inorganic salts) (Kiiyukia et al., 1993; Li et al., 1993; Kunin et al., 1995; Walker et al., 1999). Flagella were reported in several *Acidithiobacillus* species, such as *A. thiooxidans* (Doetsch et al., 1967), *A. caldus* (Hallberg and Lindstrom, 1994), and *A. albertensis* (Bryant et al., 1983; Xia et al., 2007). Different numbers and forms of flagella were observed in different species of *Acidithiobacillus*: *A. thiooxidans* and *A. caldus* cells habor monotrichous flagella while *A. albertensis* possesses lophotrichous flagella (Bryant et al., 1983; Hallberg and Lindstrom, 1994; Xia et al., 2007). However, there is limited data on flagellar molecular biology of *Acidithiobacillus*, which limits our understanding on the biofilm formation and flagella-mediated motility in these chemoautotrophic sulfur or iron oxidizers.

Flagella have been extensively studied at gene and protein levels in bacteria other than *Acidithiobacillus* spp., such as *Escherichia coli* (Kadoya et al., 2015), *Salmonella typhimurium* (Kutsukake et al., 1990; Koirala et al., 2014), *Caulobacter crescentus* (Bryan et al., 1990), *Pseudomonas aeruginosa* (Totten and Lory, 1990; Jyot et al., 2007), and *Vibrio cholerae* (Klose and Mekalanos, 1998; McCarter, 2001). The assembly and function of bacterial flagella generally require dozens of genes that encode structural subunits, regulatory proteins, motor force generators and the chemosensory system (Chen et al., 2011; Fitzgerald et al., 2014; Zhao et al., 2014; Osterman et al., 2015). These genes are organized into a large complex cluster in some bacteria, and are regulated hierarchically at transcriptional level via diverse regulatory systems (Dasgupta et al., 2000; Jacobi et al., 2004; Schulz et al., 2012; Galeva et al., 2014; Song et al., 2016). RpoF (σ^28^ or FliA) is one of the σ factors that regulates the synthesis of filaments and attracts wide attentions in various bacterial species, including *Legionella pneumophila* (Schulz et al., 2012), *E. coli* (Kundu et al., 1997), *P. aeruginosa* (Starnbach and Lory, 1992). However, the function of RpoF in *Acidithiobacillus* spp. is still not well understand.

This research reported the distribution of flagellar gene clusters in different *Acidithiobacillus* species, studied the function of the flagellum in *A. caldus* responding to unfavorable environmental conditions, and investigated the influence of RpoF on *A. caldus* transcription profile.

## Materials and Methods

### Bacteria, Plasmids, and Growth Conditions

The bacterial strains and plasmids used in this study were listed in Table 1. *E. coli* was cultivated in Luria Bertani (LB) medium with a rotation speed of 200 rpm at 37°C or plated on 1.2% agar plates (Sambrook et al., 2001). *A. caldus* MTH-04, a strain isolated from hot spring in Tengchong, was grown in modified liquid Starkey-S^0^/K_2_S_4_O_6_ media or on solid Starkey-Na_2_S_2_O_3_ plates with 1% agar at 40°C (Wang et al., 2016). Antibiotics added to LB medium include chloramphenicol (34 mg/mL), streptomycin (100 mg/mL) and kanamycin (100 mg/mL); for *A. caldus*, the concentrations of antibiotics were doubled in Starkey media. The cultivation standards of *A. caldus* MTH-04 were described earlier (Wang et al., 2016). Simply, the single colony on the solid plate was inoculated into 30 mL Starkey-S^0^ liquid medium and cultivated to stationary phase. Then, the culture was transferred into 150 mL Starkey-S^0^ liquid medium. When the culture reaches to stationary phase, cells were collected by centrifugation, and diluted with Starkey liquid medium to a final concentration of OD_600_ = 1.0. An aliquot (1 mL) of the cell solution was inoculated into 150 mL Starkey liquid medium for further experiments. Cell density was measured at OD_600_ after removal of elemental sulfur by centrifugation at 400 × *g* for 5 min (Yu et al., 2014; Wang et al., 2016).

**Table 1:** Bacteria and plasmids used in this study.

Strain	Description	Source
*A. caldus* MTH-04	Isolated from Tengchong, Yunnan Province, China	Our laboratory
WT(215)	*A. caldus* MTH-04 harboring the plasmid pJRD215	This study
WT(*rpoF*)	*A. caldus* MTH-04 harboring the plasmid pJRD215-PtetH-rpoF	This study
Δ*rpoF*	Knockout *rpoF* gene from *A. caldus* MTH-04 genome	This study
Δ*rpoF*(215)	Δ*rpoF* harboring the plasmid pJRD215	This study
Δ*rpoF*(*rpoF*)	Δ*rpoF* harboring the plasmid pJRD215-PtetH-rpoF	This study
*E. coli* SM10	Thr leu hsd recA Kmr RP4-2-Tc::Mu	Simon et al., 1983
*E. coli* DH5α	F—Φ80dlaΔM15Δ(lacZYA-argF) U169 end A1 recA1 hsdR17(rk-, mk+) supE44λ-thi-1 gyr96 relA1 phoA	TransGen Biotech Corp. China
*E. coli* BL21 (DE3)	FompT hdsSB(rB—mB—) gal dgmmet (DE3)	TransGen Biotech Corp. China
plasmids		
pJRD215	Smr, Kmr; IncQ, Mob+	Davison et al., 1987
pJRD215-PtetH-rpoF	Smr, Kmr; IncQ, Mob+; PtetH (360 bp); rpoF	This study
pSDUDI	Suicide plasmid; Apr; Kmr; oriTRP4; MCS	Our laboratory
pSDUDI-rpoF-U-D	Suicide plasmid for rpoF deletion	This study
pSDU-I-Scel	Cmr; Mob+; Rac; containing I-Scel	Our laboratory


### Molecular Biological Techniques

Taq DNA polymerase, T4 DNA ligase and restriction enzymes (Xba I, Kpn I, Nhe I and Hind III) were purchased from Takara (Kyoto, Japan). Kits for genomic DNA exaction (E.Z.N.A.^®^ Bacterial DNA Kit), plasmid extraction (E.Z.N.A.^®^ Plasmid Mini Kit I) and DNA purification (E.Z.N.A.^®^ Cycle Pure Kit) were purchased from Omega Bio-tek (Georgia, United States). Chemicals and reagents [Na_2_S_2_O_3_, K_2_S_4_O_6_, agar, (NH_4_)_2_SO_4_, KH_2_PO_4_, CaCl_2_, MgSO_4_, FeSO_4_, etc.] were purchased from Sangon (Shanghai, China) and Sinopharm (Shanghai, China). Primer synthesis and DNA sequencing were accomplished by GENEWIZ (Suzhou, China). All primers used in this study were listed in Supplementary Table S1. The genomic sequences and all sequences of flagella and chemotaxis related genes were downloaded from the public National Center for Biotechnology Information (NCBI) website https://www.ncbi.nlm.nih.gov/ (Supplementary Table S2). BlastP and PsiBlast (Altschul et al., 1997) were used to further characterize candidate genes and their predicted protein products. Syntenic regions were displayed and analyzed with modification using Genomeviz (Ghai and Chakraborty, 2007).

### Construction of *A. caldus rpoF* Mutants

The *A. caldus* MTH-04 Δ*rpoF* strain was constructed using the gene markerless knockout technique (Wang et al., 2016). The upstream flanking arm (U, 1058 bp) of the *rpoF* gene was amplified by PCR with primer set rpoF-U-F-Xba I and rpoF-U-R-Kpn I. The primer set rpoF-D-F-Kpn I and rpoF-D-R-Nhe I was used to amplify the downstream flanking arm (D, 1369 bp). Fragments U and D were digested with restriction enzymes Xba I/Kpn I and Kpn I/Nhe I, respectively. Then, these two treated fragments were ligated with the Xba I/Nhe I digested plasmid pSDUDI to obtain the suicide plasmid pSDUDI-rpoF-U-D. The resulting plasmid was verified by restriction enzyme digestion (Xba I and Nhe I) and DNA sequencing analysis before transferring into *E. coli* SM10 competent cells by heat shock transformation. Then, the suicide plasmids were conjugally transferred by filter mating from *E. coli* SM10 to *A. caldus* MTH-04 (Liu et al., 2007). The plasmid pSDUDI-rpoF-U-D was integrated into *A. caldus* MTH-04 chromosome via the homologous recombination, the single crossover mutants were selected on Starkey-Na_2_S_2_O_3_ plate containing 200 mg/mL kanamycin and determined by PCR with primer set rpoF-out-F/R. After that, the plasmid pSDU1-I-Sce I was conjugated into the single crossovers of *A. caldus*, inducing the second recombination and generating either the *rpoF* knockout mutants or wildtype cells. Primers P1 F/R, P2 F/R, and P3 F/R were designed to identify *A. caldus* Δ*rpoF* strains. The elimination of plasmid pSDU1-I-Sce I in the *rpoF* knockout strains was achieved by continuous passages (Wang et al., 2016).

The construction of *A. caldus rpoF* overexpression and complementation strains were done as follows. The fragment (rpoF) was amplified from *A. caldus* MTH-04 genome with primer set rpoF-F (containing 59-bp homologous sequence of the promoter of *tetH* gene) and rpoF-Hind III-R. The *tetH* promoter fragment (PtetH) was amplified from *A. caldus* MTH-04 chromosome with primer set PtetH-Kpn I -F and PtetH-R. The two fragments PtetH and rpoF were ligated by fusion PCR. The fused fragment PtetH-rpoF was digested with Hind III and Kpn I, and inserted into plasmid pJRD215 linearizing with the same restriction enzymes, forming the plasmid pJRD215-PtetH-rpoF. The generated plasmid was verified by sequencing and transformed into *A. caldus* wildtype and Δ*rpoF*, generating the *rpoF* overexpression strain WT(*rpoF*) and the *rpoF* complementation strain Δ*rpoF*(*rpoF*), respectively. The control plasmid pJRD215 was also conjugated into *A. caldus* wildtype and Δ*rpoF* to construct the control strains WT(215) and Δ*rpoF*(215), respectively.

### Extraction of Total RNA

For RNA extraction, experimental strains were cultivated to mid-log phase (OD_600_ 0.09–0.13) without agitation in 150 mL liquid Starkey-S^0^ media with addition of 0.4 g elemental sulfur. Cells were harvested by centrifugation (3000 × *g*, 4°C, 5 min). RNA-protect Bacteria Reagent (Qiagen) was immediately added, and pellets were stored at -80°C for backup. Total RNA was extracted with Trizol^®^ Reagent (Invitrogen) according to improved protocol’s specifications with a little modification (reaction time for each step was halved and the melting RNA was not performed). The integrity of each RNA sample was assessed by RNA formaldehyde degeneration electrophoresis in a 1.5% agarose gel with 90 mM Tris-boric acid containing 2 mM EDTA (TBE). RNA concentration and purity were determined using the NanoPhotometer^®^ spectrophotometer by measuring A260 and A260/A280 ratio. Three independent experiments were set for the purification of total RNA.

### RNA-Seq and Data Analysis

The low cell yield of *A. caldus* resulted in a small amount of total RNA in one sample, thus an aliquot of total RNA (1.0 μg) was equally taken from three biological samples of each independent experiment, and the three aliquots were mixed as one sample to perform RNA-Seq by Novogene corporation (Tianjin, China). Residual genomic DNA in the sample was digested with RNase-free DNase I (New England Biolabs). RNA concentrations were measured using Qubit^®^ RNA Assay Kit in Qubit^®^ 2.0 Flurometer (Life Technologies, CA, United States) and the integrity of these RNA samples was assessed using the RNA Nano 6000 Assay Kit of the Bioanalyzer 2100 system (Agilent Technologies, CA, United States). Then, the rRNA was removed from the mRNA using Ribo-Zero^TM^ Magnetic Kit (Bacteria). Fragmentation was carried out using divalent cations under elevated temperature in NEBNext First Strand Synthesis Reaction Buffer (5X). First strand cDNA was synthesized using random hexamer primer and M-MuLV Reverse Transcriptase (RNaseH-). Second strand cDNA synthesis was subsequently performed using DNA Polymerase I and RNase H. In the reaction buffer, dNTPs with dTTP were replaced by dUTP. Remaining overhangs were converted into blunt ends via exonuclease / polymerase activities. After adenylation of 3′ ends of DNA fragments, it was ligated to NEBNext Adaptor with hairpin loop structure for hybridization. In order to select cDNA fragments of preferentially 150∼200 bp in length, the library fragments were purified with AMPure XP system (Beckman Coulter, Beverly, NJ, United States). Then 3 μl USER Enzyme (NEB, United States) was applied on size-selected, adaptor-ligated cDNA at 37°C for 15 min followed by 5 min at 95°C before PCR. Then PCR was performed with Phusion High Fidelity DNA polymerase, Universal PCR primers and Index (X) Primer. At last, products were purified (AMPure XP system) and library quality was assessed on the Agilent Bioanalyzer 2100 system. The clustering of the index-coded samples was performed on a cBot Cluster Generation System using TruSeq PE Cluster Kit v3-cBot-HS (Illumia) according to the manufacturer’s instructions and sequenced using an Illumina Hiseq platform.

Raw data (raw reads) of fastq format were firstly processed through in-house perl scripts. Clean data (clean reads) were obtained by removing reads containing adapter, the pair-end reads satisfied *N* > 10%, and low *Q*-value reads [(quality value < 20) > 50%] from raw reads. Clean reads were mapped to the *A. caldus* MTH-04 genome (Accession number: LXQG00000000). HTSeq v0.6.1 was used to count the read numbers mapped to each gene. And then the FPKM (expected number of Fragments Per Kilobase of transcript sequence per Millions base pairs sequenced) of each gene was calculated based on the length of the gene and the number of the reads mapped to this gene (Trapnell et al., 2009).

The read counts were adjusted by edgeR program package through one scaling normalized factor before differential gene expression analysis (Wang et al., 2010). Differential expression analysis was performed using the DEGSeq R package (1.20.0). The *P*-values were adjusted using the Benjamini and Hochberg method (Benjamini and Hochberg, 1995; Klipper et al., 1995). Corrected *P*-value of 0.005 and log_2_FC (Fold change) of one were set as the threshold for significantly differential expression.

Gene Ontology (GO) enrichment analysis of differentially expressed genes (DEGs) was implemented by the GOseq R package (Young et al., 2010), in which gene length bias was corrected. GO terms with corrected *P*-value less than 0.05 were considered significantly enriched by differential expressed genes. KOBAS software was used to test the statistical enrichment of DEGs in KEGG pathways.

The raw data of RNA-seq is deposited in NCBI with accession number PRJNA528607. And original analysis of DEGs is listed in Supplementary Table S3.

### Real-Time Quantitative PCR (RT-qPCR)

The removal of gDNA and reverse transcription of the RNA samples were performed using PrimeScript^TM^ RT Reagent Kit (Takara) according to instructions. Quantitative amplification was performed with SYRB^®^ Premix Ex Taq^TM^ (Takara) on the Roche LightCycler 480 system (Roche). The *gapA* gene, encoding glyceraldehyde-3-phosphate dehydrogenase, was used as reference gene for normalization of RT-qPCR data (Chen et al., 2013). The 2^-ΔΔCt^ method was used to analyze relative changes in gene expression, where [ΔCt = (Ct *_target_* – Ct _gapA_) _mutantorwildtype_, ΔΔCt = ΔCt _mutant_ – ΔCt _wildtype_] (Livak and Schmittgen, 2001; Li et al., 2017). Each sample was repeated with three independent extractions of RNA and the standard deviations were calculated and showed as error bars. All primers designed are listed in Supplementary Table S1.

### Transmission Electron Microscope (TEM)

Bacteria were inoculated on Starkey-Na_2_S_2_O_3_ plate and grown for 8 days. The colony was gently picked out with blunt tip and suspended in water. This suspension was added dropwise on grids for several minutes, then stained with 2% tungstophosphoric acid for a few seconds before being observed under a Quanta FEG 250 (FEI) electron microscope.

### Scanning Electron Microscope (SEM)

Sulfur (S^0^) coupons were produced by heating elemental sulfur powder until melting in a fume hood. Subsequently, the liquid sulfur was poured on a cover glass and cooled. Coupons were sterilized in an autoclave at 103°C for 3 h (Gonzalez et al., 2013). The initial cells of *A. caldus* prepared according to standard cultivation process (Wang et al., 2016), were equally inoculated into 150 mL Starkey media stuffing 5 g S^0^ coupons, and then cultivated for another 7 days with or without agitation and other conditions as usual. Sulfur coupons from the culture were dehydrated through a graded ethyl alcohol series and dried with the critical point drier. Specimens were mounted on stubs, coated with gold, and examined under a FEI Quanta250 FEG at 10 kV. Three coupons and five fields were imaged for each sample.

### Swarming Assay

*Acidithiobacillus caldus* cells were grown for 7 days, and cells were collected by centrifugation (10000 × *g*, 20°C, 5 min). The bacterial suspension was diluted to an OD_600_ of 0.5, then punctured into semi-solid medium (Starkey-Na_2_S_2_O_3_ medium containing 0.3% agar) and incubated under aerobic conditions at 40°C. Motility was estimated as the diameter of colonies. The areas of at least 12 colonies grown in three independent plates were measured by Image J software (Collins, 2007).

## Results

### Distribution of Flagellar Encoding Genes in Different Species of *Acidithiobacillus*

**FIGURE 1:**
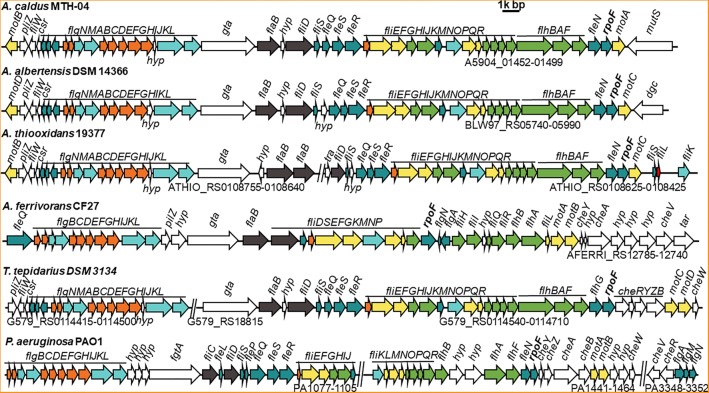
Comparison of flagellar gene clusters among different *Acidithiobacillus* species and *Pseudomonas aeruginosa.* GenBank accession numbers for these genomes and the gene IDs for corresponding flagellar cluster are, *A. caldus* MTH-04 (NZ_CP026328, A5904_01452-01499); *A. albertensis* DSM 14366 (NZ_MOAD01000001, BLW97_RS06020-RS05990); *A. thiooxidans* 19377 (NZ_AFOH01000122, ATHIO_RS0108425-RS0108625, ATHIO_RS010860-RS0108755); *A. ferrivorans* CF27 (NZ_CCCS020000023, AFERRI_RS12740-RS12785); *Thermithiobacillus* tepidarius DSM 3134 (NZ_KE384096, G579_RS0114415-0114500, G579_RS18815, G579_RS0114540-0114710); *Pseudomonas aeruginosa* PAO1 (NC_002516, PA1077-1105, PA1441-1464, PA3348-3352). The annotation of each gene is listed in Supplementary Table S2. Different colors mean different structural component of flagellum and other related function: filament is in dark gray, hook is in aquamarine, external basal body (L- and P-ring) is in orange, inner basal body (MS- and C-ring) is in yellow, green represents export apparatus, teal represents regulators and chaperones and white stands for chemotaxis and other unknown function proteins.

On basis of their energy substrates, *Acidithiobacillus* species can be grouped into the sulfur oxidizers (*A. caldus*, *A. thiooxidans*, and *A. albertensis*) and the iron/sulfur oxidizers (*A. ferrooxidans*, *A. ferridurans*, *A. ferriphilus*, and *A. ferrivorans*). Results showed that all the sulfur oxidizers in *Acidithiobacillus* harbor the flagellar biosynthesis related genes (Figure 1 and Supplementary Tables S2, S4). However, that is not always the case for the sulfur / iron oxidizers of *Acidithiobacillus* (Supplementary Table S4). Flagellar gene cluster is discovered in *A. ferrivorans*, one of the sulfur / iron oxidizer species, but not found in either *A. ferrooxidans* or *A. ferridurans*. Three *A. ferrivorans* strains (CF27, PRJEB5721, and YL15) have flagellar gene clusters that are different from those in the sulfur oxidizers of *Acidithiobacillus* (Figure 1 and Supplementary Table S4). In contrast, the flagellar gene cluster is not found in the published genomes of other three strains of *A. ferrivorans* (SS3, 21-59-9 and PQ33) (Supplementary Table S4). The absence of the flagellar gene cluster in *A. ferrivorans* SS3 complete genome suggests that the flagella might not be present in all strains of *A. ferrivorans*, but not in *Acidithiocillus* spp. lacking the Sox system. To be specific, the flagellar gene clusters are found in the species that possess the Sox system, such as *A. caldus*, *A. thiooxdans*, *A. ferrivorans*, *A. albertensis*, and *Thermithiobacillus tepidarius* (Figure 1), but not in strains lacking the Sox system in *A. ferrooxidans* or *A. ferridurans* (Supplementary Tables S2, S4). Flagellar gene clusters are also found in iron oxidizers used in bioleaching, such as *Leptospirillum ferrooxidans* C2-3 and *Leptospirillum. ferriphilum* ML-04 (Supplementary Figure S6).

All flagellar biosynthesis related genes in *A. caldus* and *A. albertensis*, including flagellin genes, regulatory genes and chemotactic genes, are arranged together in one large gene cluster, but these genes are separated into two gene clusters in *A. thiooxdans* and *T. tepidarius* DSM3134. Although a flagellar gene cluster is found in *A. ferrivorans*, the genes and their arrangement orders in *A. ferrivorans* genomes are distinct from that of *A. caldus*. No other genes were found in this cluster in *A. caldus* except for one gene *gta*, encoding a glycosyl transferase that locates between the basal body/hook gene cluster and the filament gene cluster and is considered to be associated with the glycosylation of flagellins (Figure 1; Arora et al., 2005). In contrast, the flagellar genes of *P. aeruginosa*, *V. cholera*e, and *E. coli* are separated into at least three loci in the genome by other unrelated genes (Figure 1 and Supplementary Figure S6).

FliC is shown to have pleiotropic effect on bacterial motility, growth, biofilm formation and protein secretion (He et al., 2012). The absence of FliC led to the absence of flagella and the deficiency of motility for the cells (Tremblay et al., 2012). There are five filament related genes (*flaAC* and *flaEDB*) found in the genome of *V. cholerae* (Prouty et al., 2001), however, only *flaB* (a *fliC* homologous gene) was found in *A. caldus*. Sequence analysis indicates that FlaB has 31.5% identity to FliC of *E. coli*, but they differ in immunostimulatory properties (Lee et al., 2003). Besides, phylogenetic analysis indicates that the FlaB protein from *Acidithiobacillus* was closely related to that of *Thiomonas* family (Supplementary Figure S1). Interestingly, FlhDC, essential regulators for flagella biosynthesis in *E. coli* (Wang et al., 2006; Lee et al., 2011), are found in none of *A. caldus*, *A. albertensis*, and *A. thiooxdans*. In contrast, a σ^28^ factor (RpoF or FliA) and four σ^54^-dependent regulatory proteins (FleQ, FleR, FleS, and FleN) are encoded in the genome of these chemoautotrophic sulfur or iron oxidizers. The flagellar alternative sigma factor RpoF is located at the end of the flagellar gene clusters in *Acidithiobacillus* strains and shows 46.9% identity to the RpoF in *E. coli* and 48.43% to that of *P. aeruginosa*.

### Construction of Different *A. caldus rpoF* Mutants

**FIGURE 2:**
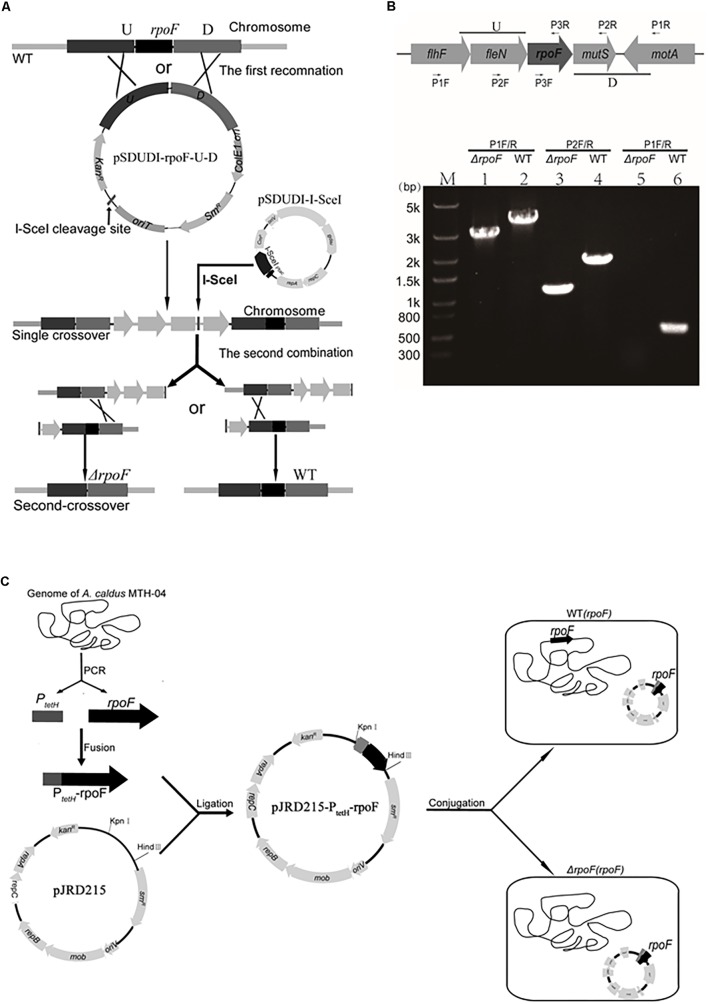
Construction of *rpoF* deletion, overexpression and complementation strains. **(A)** The schematic of *rpoF* double-crossover markerless gene deletion system. **(B)** Confirmation of *rpoF* mutant by PCR. Positions of the three sets of primers used for PCR amplifications to confirm *rpoF* mutagenesis. U, upstream homologous arm of *rpoF*; D, downstream homologous arm of *rpoF*. Confirmation of *rpoF* knockout strain by PCR analyses. *flhF* (flagellar biosynthesis regulator FlhF), *fleN* (Cobyrinic acid a,c-diamide synthase), *rpoF* (flagellar biosynthesis sigma factor), *motA* (flagellar motor protein MotA) and *mutS* (DNA mismatch repair protein). Lanes 1, 3, and 5, PCR amplifications from *A. caldus* Δ*rpoF* genome with primers P1F/R, P2F/R and P3F/R, respectively. Lanes 2, 4, and 6, PCR fragments amplified from the genome of wildtype strain with primers P1F/R, P2F/R and P3F/R, respectively. **(C)** Construction of *rpoF* overexpression and complementation strains. WT(*rpoF*), wildtype strain harboring plasmid pJRD215-P_tetH_-rpoF; Δ*rpoF*(*rpoF*), *rpoF* knockout strain containing plasmid pJRD215-P_tetH_-rpoF (Liu et al., 2007).

Different *rpoF* mutants were constructed to reveal the role of RpoF in regulating flagellar biosynthesis in *A. caldus* MTH-04. An “in-out” gene markerless deletion strategy (Figure 2A) was used to generate *A. caldus rpoF* deletion strain as described in the “Materials and Methods” section. The 2976-bp and 1111-bp fragments were amplified from *A. caldus* Δ*rpoF* strain using primer sets P1 F/R specific to the lateral regions of homologous arm and P2 F/R specific to the homologous arm regions, respectively (Figure 2B, lanes 1 and 3). Larger fragments were obtained from *A. caldus* wildtype with the two primer sets (3,663-bp for P1 F/R and 1,824-bp for P2F/R) (Figure 2B, lanes 2 and 4). No band was detected in *A. caldus* Δ*rpoF* strain using primers (P3F/R) specific to *rpoF* gene (Figure 2B, lane 5), while a 543-bp fragment was amplified from wildtype strain (Figure 2B, lane 6). PCR fragments amplified from genome of *A. caldus* Δ*rpoF* strain using primers P1F/R were sequenced to make sure that *rpoF* gene was markerlessly removed from *A. caldus* MTH-04 chromosome. A *rpoF*-expression plasmid was constructed successfully using the mobilizable plasmid pJRD215 as backbone (Figure 2C). The promoter (P_tetH_) of tetrathionate hydrolase gene (*tetH*) of *A. caldus* was selected to induce the transcription of *rpoF* (Figure 2C). The *A. caldus rpoF* complemented strain [Δ*rpoF*(*rpoF*)], the overexpression strain [WT(*rpoF*)] and control strains [Δ*rpoF*(215) and WT(215)] were obtained as described in “Materials and Methods” section (Figure 2C).

### RpoF Regulates Flagellar Biosynthesis and Cell Motility of *A. caldus*

**FIGURE 3:**
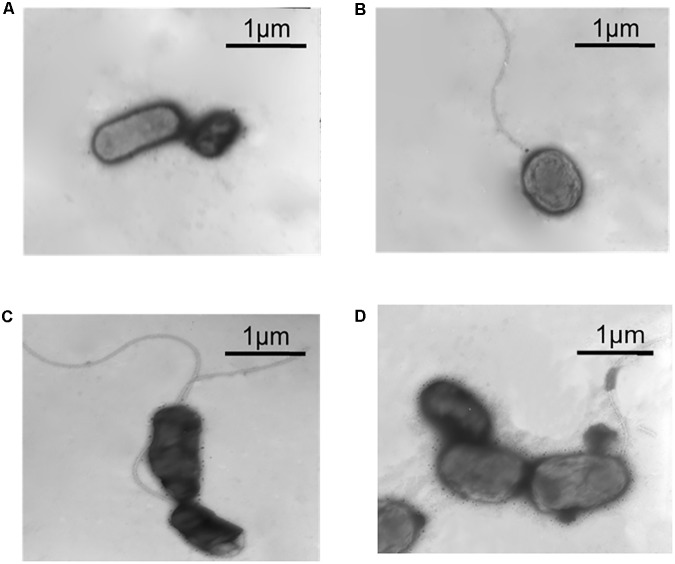
Observation of different *A. caldus* strains using transmission electron microscope (TEM). **(A)** *A. caldus* Δ*rpoF*(215) strain, the *rpoF* deletion strain containing blank plasmid pJRD215. **(B)** *A. caldus* Δ*rpoF*(*rpoF*) strain, the Δ*rpoF* complemented with the *rpoF* gene using the plasmid pJRD215-P_tetH_-rpoF. **(C)** *A. caldus* WT(215) strain, the wildtype strain harboring pJRD215. **(D)** *A. caldus* WT(*rpoF*) strain, the overexpression of *rpoF* gene in *A. caldus* wildtype using pJRD215-*P_tetH_-rpoF*.

**FIGURE 4:**
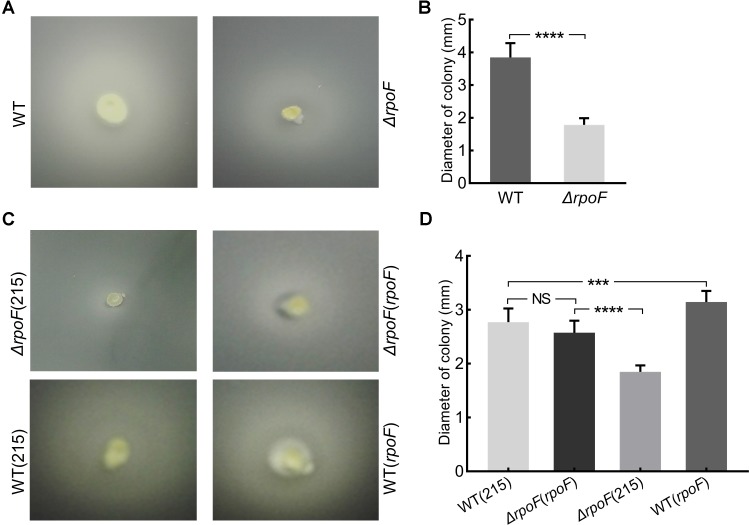
Motility analysis of *A. caldus rpoF* mutants. **(A,C)** Colonies of different strains on the semi-solid agar. **(B,D)** statistical analysis on the swarming diameters. Graphpad Prism7.0 was used to measure the swarming diameters of at least 20 colonies for each strain. The error bars showed Standard Error of the Mean (SEM). The significance was determined using Student’s *t*-test. NS, no significance; ^∗∗∗^*P* < 0.001, significant; ^∗∗∗∗^*P* < 0.0001, extremely significant. WT, *A. caldus* wildtype; Δ*rpoF*, the *rpoF* deletion strain; Δ*rpoF*(215), the *rpoF* deletion strain carrying blank plasmid pJRD215; Δ*rpoF*(*rpoF*), the *rpoF* deletion strain complemented with plasmid pJRD215-*P_tetH_-rpoF*; WT(215), the wildtype strain harboring pJRD215; WT(*rpoF*), the overexpression of *rpoF* gene in *A. caldus* wildtype strain using pJRD215-P_tetH_-rpoF.

Transmission electron microscope was used to observe the flagella of different *A. caldus* mutants. No flagellum was found in *A. caldus* Δ*rpoF* strain, whereas a single polar flagellum was observed in the wildtype strain, the *rpoF* complementation strain and the overexpression strain of *A. caldus* (Figure 3). The result indicates that *rpoF* is essential for flagellar biosynthesis in *A. caldus*. Further, the motility of these *A. caldus* strains was investigated via the swarming assay. The swarming diameters of *A. caldus* wildtype were approximately twice as large as those of Δ*rpoF-*formed colonies on semi-solid media (Figure 4A,B). This result suggests that the motility of *A. caldus* Δ*rpoF* strain is inhibited significantly. No significant differences were detected between the *rpoF* complementation strain Δ*rpoF*(*rpoF*) and the control strain WT(215) in terms of the swarming diameters (Figure 4C,D). *A. caldus rpoF* overexpression strain WT(*rpoF*) showed an increase in colony diameters compared with that of the control strain WT(215) (Figure 4C,D). Thus, the deletion or overexpression of *A. caldus rpoF* leads to visible effects on the size of colonies, indicating the importance of flagella to *A. caldus* motility.

### RpoF Influences the Growth of *A. caldus* in Unfavorable Conditions

**FIGURE 5:**
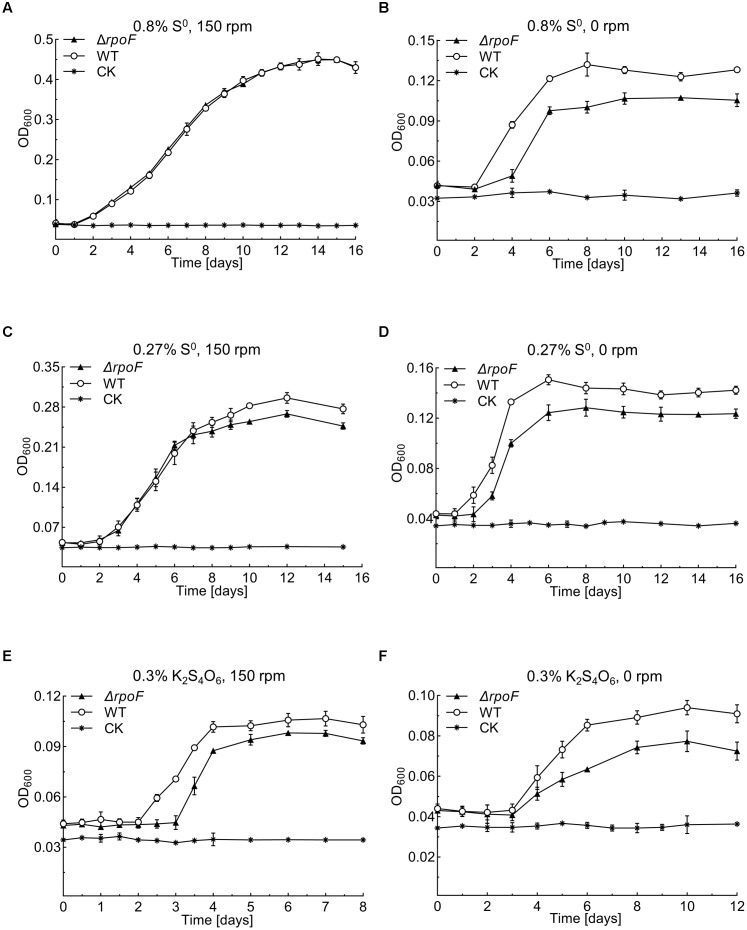
Growth curves of the *A. caldus* MTH-04 wildtype and Δ*rpoF* under different growth conditions. All measurements were performed in triplicates. GraphPad Prism 7.0 was used for statistical analysis. **(A,B)** The growth of strains in Starkey-S^0^ medium with the addition of 1.2 g S^0^ at 150 rpm and 0 rpm, respectively. **(C,D)** The growth of strains in Starkey-S^0^ medium with the addition of 0.4 g S^0^ at 150 rpm and 0 rpm, respectively. **(E,F)** The growth of strains in Starkey-K_2_S_4_O_6_ medium containing 0.1 mM K_2_S_4_O_6_ at 150 rpm and 0 rpm, respectively. CK, control check, medium without cells.

Flagella play essential roles in bacterial chemotaxis and motility thus this sophisticated apparatus facilitates the survival of bacteria in various complex environments (Arora et al., 1997; McGowan et al., 2000; Shah et al., 2000; Young et al., 2000; Jahn et al., 2008; Koster et al., 2012; Stevenson et al., 2015). To investigate the influence of RpoF absence on *A. caldus* growth, we created several unfavorable conditions, including static cultivation, low concentration of S^0^ and low-energy-density substrate (S_4_O_6_^2-^). When *A. caldus rpoF* deletion and wildtype strains were cultivated in the optimal growth conditions (150 rpm, 1.2 g S^0^ added in 150 mL media), no growth differences were observed in either their growth curves or growth rates (Figure 5A and Supplementary Figure S2A). When the cells were cultivated without agitation, *A. caldus* Δ*rpoF* showed an obvious longer lag phase and lower OD_600_ value compared to those of wildtype strain (Figure 5B). The OD_600_ of wildtype and mutant strains reached 0.135 (±0.009) and 0.104 (±0.004) at the stationary phase, respectively (Figure 5B), and *A. caldus* wildtype strain showed an advantage on the growth rate over that of the mutant before entering stationary phase (Supplementary Figure S2B). These results indicate that agitation is an important factor for the growth of *A. caldus ΔrpoF.* When the amount of elemental sulfur was decreased to 0.27% (weight/volume) (0.4 g S^0^ per 150 mL), the difference in growth was detected not only at static condition but also at the shaking condition (Figure 5C,D and Supplementary Figures S2C,D). With agitation and 0.4 g S^0^, the growth disadvantage and lower growth rate of *A. caldus* Δ*rpoF* were observed in the stationary phase (Figure 5C and Supplementary Figure S2C), indicating the limited energy-substrate had a negative effect on the growth of *rpoF* mutant under shaking condition. When tetrathionate was used as the energy-substrate, *A. caldus* Δ*rpoF* exhibited much lower cell density and growth rate compared with that of wildtype strain regardless of agitation or stilling cultivation (Figure 5E,F and Supplementary Figures S2E,F). As a result, the absence of RpoF has no obvious influence on *A. caldus* growth under optimal conditions (1.2 g S^0^ and 150 rpm), but brings negative effects on its growth in unfavorable conditions (no shaking, low substrate concentration or non-optimal substrate).

The pH value is an important environmental factor that affects the growth of *A. caldus* on elemental sulfur. Under the optimal condition of 1.2 g S^0^ and 150 rpm, the pH of both *A. caldus* Δ*rpoF* and wildtype cultures declined from 2.5 (in the 1st day) to approximately 0.7 (in the 9th day) (Supplementary Figure S3A). When the amount of S^0^ was decreased to 0.4 g in shaking condition, the pH of wildtype culture dropped to 0.97, which is obviously lower than the pH of *A. caldus* Δ*rpoF* culture (1.12) (Supplementary Figure S3B). Under the static cultivation, *A. caldus* Δ*rpoF* culture containing 0.4 g or 1.2 g S^0^ showed higher pH values than that of wildtype strain, but the final pH of all these cultures was above 1.0 (Supplementary Figures S3C,D). Therefore, the change patterns of pH values of *A. caldus* Δ*rpoF* and wildtype strain cultures were similar to that of cell growth, indicating the correlation between cell growth and the environmental pH changes.

### The Absence of RpoF Affects *A. caldus* Attachment

**FIGURE 6:**
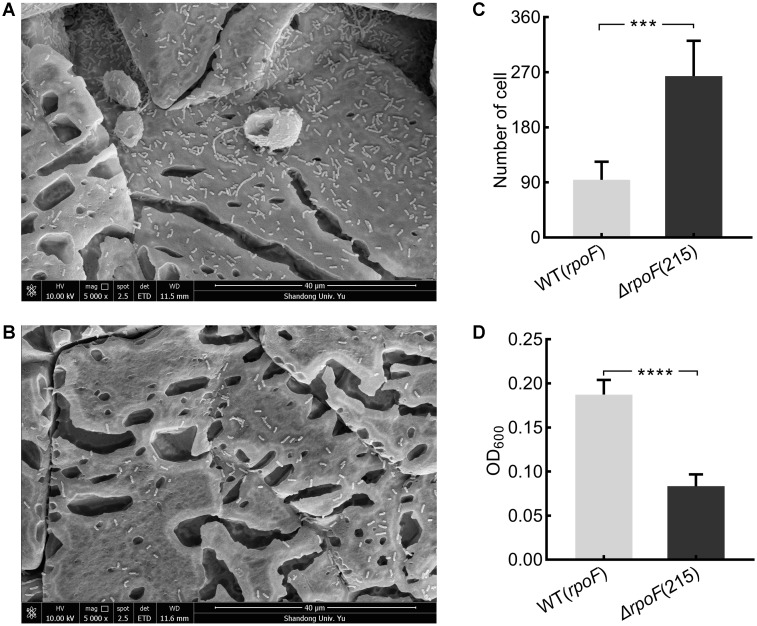
The distribution of *A. caldus rpoF* deletion and overexpression strains on the S^0^ coupons. Scanning electron micrographs for strains Δ*rpoF*(215) **(A)** and the *rpoF* overexpression [WT(*rpoF*)] **(B)** on sulfur-coupons in static cultivation. Statistical analysis on the number of cells from five different microscopic fields on S^0^ coupon for Δ*rpoF*(215) and the *rpoF* overexpression [WT(*rpoF*)] strain **(C)**. The OD_600_ of free cells in liquid Starkey-S^0^ coupon containing media for Δ*rpoF*(215) and WT(*rpoF*) in five duplicates **(D)**. ^∗∗∗^*P* < 0.001, significant, ^∗∗∗∗^*P* <0.0001, extremely significant.

To investigate the effects of flagella on cell attachment, *A. caldus* MTH-04 *rpoF* deletion and overexpression strains were cultivated with S^0^ coupons (see section “Material and Methods”). Under the static condition, flagella-absent cells tended to attach on the S^0^ coupon surface (Figure 6A–C), while cells with flagella were inclined to be in the free-living state and the cell density of the *rpoF* overexpression strain was three times higher than that of the *rpoF* deletion strain in the liquid culture (Figure 6D). Results indicate that the absence of RpoF results in the tendency of cells to attach to S^0^-coupons. When the cultivation condition was changed to agitation, no significant differences in cell numbers were detected between the two strains on S^0^ coupons (Supplementary Figure S4).

### The Influence of RpoF Mutagenesis on the Transcription of Flagellar Biosynthesis Gene Cluster and the Transcriptome Profiling of *A. caldus*

**FIGURE 7:**
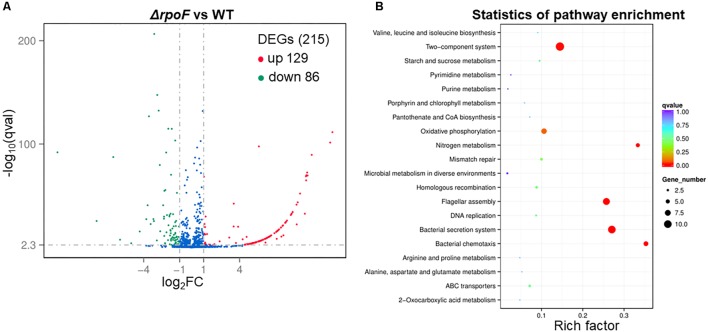
Overall transcriptomic changes of Δ*rpoF*. **(A)** Volcano plot showing fold changes and levels of significance for differential expression genes. **(B)** KEGG pathway enrichment.

To explore the regulatory role of RpoF, *A. caldus* Δ*rpoF* and wildtype cells in static cultivation were collected at mid-log growth phase to perform RNA-seq. There were 215 DEGs in *A. caldus rpoF* knockout strain compared with wildtype strain, including 129 upregulated genes and 86 downregulated genes (Figure 7A and Supplementary Table S3). Forty-one DEGs were selected to perform RT-qPCR assays and the fold changes of these genes were calculated. Statistical analyses of the fold change values from RT-qPCR and RNA-seq indicate that 39 of the 41 genes located in 95% confidence limit (Supplementary Figure S5). The high consistency of the data from RNA-seq and RT-qPCR suggests that the RNA-seq data are reliable. The KEGG pathway enrichment of the DEGs indicates that 20 pathways were influenced by the deletion of *rpoF* in *A. caldus*, referring to flagellar assembly, bacterial chemotaxis, section system, two-component system, nitrogen metabolism, oxidative phosphorylation, DNA modification, etc. (Figure 7B and Supplementary Table S5). These results suggest that RpoF plays an important role in regulating the transcription profile of *A. caldus*.

The deletion of *rpoF* in *A. caldus* MTH-04 resulted in significant downregulation of some genes involved in flagellar synthesis, including *fliW* (A5904_1454), *csrA* (A5904_1455), *flavoprotein* (A5904_1456), *hyp* (A5904_1457), *hyp* (A5904_1469), *flgK* (A5904_1470), *flgL* (A5904_1471), *fliC* (A5904_1473), *hyp* (A5904_1474), *fliD*(A5904_1475), *fliS* (A5904_1476), *fliA* (A5904_1498), *fliL* (A5904_1536), *motA* (A5904_1499), and *motD* (A5904_1452) (Supplementary Table S5). Thus, the regulation of RpoF on the transcription of flagellar gene cluster in *A. caldus* was verified here. The genes are responsible for the chemotaxis such as *cheW* (A5904_1445), *cheA* (A5904_1448), *cheZ* (A5904_1449), and *cheY* (A5904_1450), showed different degrees of downregulation in *A. caldus rpoF* deletion strain (Supplementary Table S5). The low expression levels of these genes in *A. caldus rpoF* knockout strain implies the close connections between RpoF and chemotaxis in this bacterium. In summary, RpoF controls the expression of flagellar genes, which explains why Δ*rpoF* does not assemble the flagellin at the transcriptional level. The extremely low transcriptional levels of some genes [A5904_0005 (extracellular solute-binding protein), A5904_0969 (diguanylate cyclase/phosphodiesterase with PAS/PAC and GAF sensor), and A5904_1537 (conserved hypothetical protein)] indicate that the transcription of these genes may be RpoF-dependent.

The deletion of *rpoF* gave rise to transcriptional changes of genes participating in energy metabolism and respiratory chain in *A. caldus*. Different types of terminal oxidase encoding genes showed distinct transcriptional changes: the cytochrome c oxidase genes *cydB* (A5904_1252), *cydA* (A5904_1253), *ccdA* (A5904_1799), NADH dehydrogenase genes *nuoB* (A5904_0714) and *ndhF* (A5904_2209) were downregulated, while the NADH-ubiquinone oxidoreductase genes *nuoG* (A5904_0764), *nuoH* (A5904_0765), *nuoI* (A5904_0766), and *nuoN* (A5904_0771) were upregulated (Supplementary Table S5). The transcriptional differences of these terminal oxidase encoding genes indicate that the absence of RpoF could influence the electron transfer in *A. caldus*. However, almost none of the canonical sulfur-oxidizing genes exhibited transcriptional changes after *rpoF* mutagenesis in *A. caldus*, indicating deleting *rpoF* has no effect on the typical sulfur metabolism (Sox system, S_4_I pathway, and Dsr system). The influence of RpoF mutagenesis on the other cellular metabolisms was discovered, including the downregulation of genes in the nitrogen metabolism and upregulation of genes in the carbon metabolism (Supplementary Table S5). Unusually, A5904_0981 (glucan 1,4-alpha-glucosidase) and A5904_0114 (bactoprenol glucosyl transferase) were upregulated more than 400 times in *A. caldus* Δ*rpoF* strain, suggesting RpoF plays an important role in these corresponding biological processes (energy, nitrogen, and carbon metabolisms).

The absence of RpoF in *A. caldus* MTH-04 also led to different influences on transcription of genes related to cell permeability and secretion systems. Transporter genes, including A5904_0100 (major facilitator superfamily MFS1), A5904_0983 (O-antigen ABC transporter), A5904_2283 (ExbB proton channel), A5904_2356 (potassium channel protein), A5904_2775 (major facilitator superfamily MFS1), and A5904_2774 (nitrate transporter), exhibited lower transcription levels in the *rpoF* knockout strain, while 21 conjugation related genes were upregulated in this mutant and some of them even increased more than 100 times at transcription level (Supplementary Table S5). The transcription of genes in type IV secretion system, such as A5904_1857-1865 (in the order of *pilN*, *pilO*, *pilP*, *pulE*, *pulF*, *pilS*, *pilU*, *pilM*, and *pilV*), was upregulated in *A. caldus* Δ*rpoF* strain. However, as for pilus synthetic genes, A5904_0039 (*pilZ*), A5904_0041 (*pleD*), A5904_0042 (conserved hypothetical protein), and A5904_0043 (prepilin-type N-terminal cleavage/methylation domain-containing protein) were downregulated in *A. caldus rpoF* deletion strain (Supplementary Table S5).

Some of the transcription regulator genes including a σ factor genes *rpoE* (A5904_0065), Fis family transcriptional regulator (A5904_1002), LysR family transcriptional regulator (A5904_2210), and two component systems [*cheA/Y*, *cydB/C* (A5904_1252/1253)] were downregulated, while some genes like A5904_1597 (helix-turn-helix XRE-family like protein), A5904_2806 (helix-turn-helix XRE-family like protein), and A5904_0628 (looped-hinge helix DNA binding domain, AbrB family) were upregulated in *A. caldus* Δ*rpoF* strain (Supplementary Table S5). The similar transcriptional changes were also present among transposase genes. Besides, the DNA replication and modification were affected by the deletion of *A. caldus rpoF* as well (Supplementary Table S5).

## Discussion

In this study, we discovered that the flagellar gene clusters were distributed in almost all *Acidithiobacillus* species (Supplementary Table S4) with two exceptions, *A. ferrooxidan* (Valdés et al., 2008b) and *A. ferridurans*. The sulfur oxidizers that harbor the Sox system tend to form the flagella. Sox system, consists of SoxXA, SoxYZ, and SoxB in *Acidithiobacillus* spp., is an important and highly efficient pathway for these sulfur oxidizers to obtain electrons for ATP production (Mangold et al., 2011; Chen et al., 2012; Yin et al., 2014; Christel et al., 2016; Wang et al., 2019). The existence of Sox system in these flagella-contained strains in *Acidithiobacillus* implies the importance of energy supply in the occurrence of flagella in *Acidithiobacillus* strains (Figure 1 and Supplementary Table S4). Similar rules also applied to *Thiobacillus* spp. (Supplementary Table S4) which are phylogenetically close to *Acidithiobacillus* spp.

**FIGURE 8:**
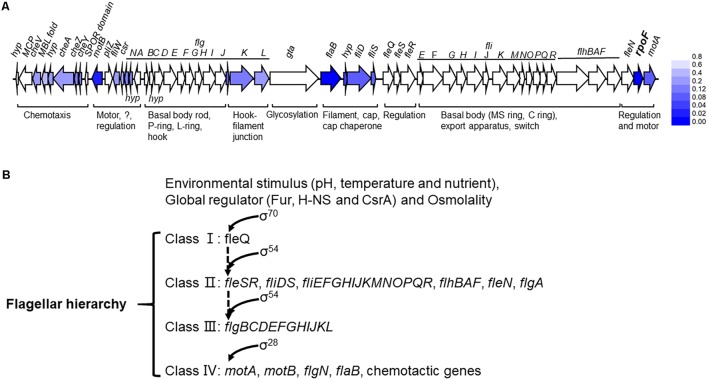
The flagellar transcription hierarchy model of *A. caldus*. **(A)** The influence of RpoF on the transcription of flagellar genes. The blue color stands for the downregulation of gene in *A. caldus* Δ*rpoF* and the darker color means the greater difference; **(B)** the proposed model of flagellar regulation hierarchy in *A. caldus*.

The flagellum of *A. caldus* is highly similar to that of *P. aeruginosa* in morphological structure (McCarter, 2006; Potvin et al., 2008) in addition to the similar flagellar gene arrangement and flagellar regulatory proteins (Figure 1 and Supplementary Table S2). The regulatory role of RpoF for flagellar assembly in *A. caldus* MTH-04 was determined by TEM observation (Figure 3) and RNA-seq analysis (Figure 8A). These findings indicate that *A. caldus* probably develops the similar flagellar transcription hierarchy as the reported pattern in *P. aeruginosa* (Ritchings et al., 1995; Dasgupta et al., 2002, 2003; Soutourina and Bertin, 2003; Luo et al., 2016). As shown in Figure 8B, the flagellar biosynthesis in *A. caldus* is probably controlled by a four-class hierarchy involving the regulatory proteins RpoN, FleQ, FleS, FleR, and RpoF (FliA). The only Class I gene is *fleQ* which encodes a σ^54^-dependent activator that is transcribed by a σ^70^ promoter. FleQ, along with σ^54^-holoenzyme, could modulate the transcription of Class II flagellar genes including *fleSR*, *fliDS*, *fliEFGHIJKMNOPQR*, *flhBAF*, *fleN*, and *flgA*. The Class II genes encode flagellar structural components of basal body (MS ring and C ring), export apparatus, switch, as well as the regulatory factors (FleS/FleR, FleN, RpoF, and FlgA). FleS/FleR, a σ^54^-dependent two component regulatory system (TCS) in which FleS is a sensor kinase for the response regulator, FleR, could regulate the transcription of the Class III genes (*flgBCDEFGHIJKL*) which encode flagellar basal body rod, P-ring, L-ring and hook. The Class IV genes are σ^28^-dependent, and are responsible for encoding the alternative flagellins, the putative anti-sigma factor FlgM, the motor components MotAB and chemotactic protein (CheY, CheZ, and CheA).

Flagella-driven bacterial motility and chemotaxis are important for microorganisms to find energy substrates and respond to unfavorable environments (Prouty et al., 2001; Soutourina and Bertin, 2003; Liu et al., 2005; Seshasayee et al., 2006; Jonas et al., 2010). A conclusion is drawn from the growth analysis of *A. caldus* MTH-04 (Figure 5), that flagella endows *A. caldus* with a strong ability to adapt to the unfavorable environment. Previous works reported that cells still managed to assign the energy for flagella synthesis and assembly even in the unfavorable environments (Liu et al., 2005; Zhao et al., 2007). Wildtype cells of *A. caldus* grew better than the Δ*rpoF* in unfavorable conditions (static cultivation or low concentration of energy-substrates) (Figure 5), indicating cells could benefit from flagellar biosynthesis even though it is an energy-consuming process. Recently, an evolutionary tuning of the trade-off between flagella-driven motility and growth proposed that bacterial cells tend to synthesize flagella to gain a better chance for survival when the substrates are insufficient (Ni et al., 2017). The weaker growth of Δ*rpoF* when little energy substrates were provided (0.4 g, Figure 5C,D) suggests that this trade-off model may apply to chemoautotrophic bacteria as well.

Flagella-mediated cell motility affects the lifestyles of *A. caldus* in the S^0^ coupon culture. Under static cultivation conditions, cells with flagella are motile (Figure 4) so that cells tended to be planktonic for easily gaining the energy substrates from solid surfaces and obtaining the oxygen and carbon dioxide from the liquid phase for growth (Figure 6B,D). In contrast, the absence of flagella made the cells immotile (Figure 4), resulting in the tendency of cells attaching on the surface of S^0^ coupon (Figure 6A,C). When *A. caldus* strains were cultivated with agitation, it was easy for cells to contact the energy-substrate and air, thus the loss of motility caused by absence of RpoF could not lead to the cell-attachment difference in the S^0^ coupon cultivation (Supplementary Figure S4). Therefore, the flagella could allow *A. caldus* to better respond to the environmental stress and obtain growth advantage in the unfavorable condition.

The absence of RpoF influenced the transcription of genes involved in *A. caldus* biofilm formation. Type IV pili is reported to be responsible for biofilm formation (O’Toole and Kolter, 1998; Pratt and Kolter, 1998; Li et al., 2010; Giltner et al., 2012). Genes of type IV pili (A5904_1858 – 1865) were significantly upregulated in *A. caldus rpoF* knockout strain, implying the flagella-absent cells are likely to form biofilm. Recent works have shown that the second messenger cyclic diguanylate (c-di-GMP) is another central regulator for biofilm formation in bacteria (Boyd and O’Toole, 2012; Purcell et al., 2012; Romling et al., 2013; Purcell and Tamayo, 2016), and c-di-GMP also plays an important role in the adhesion of *Acidithiobacillus* spp. to solid surfaces (Ruiz et al., 2012; Castro et al., 2015; Diaz et al., 2018). Several genes related to the c-di-GMP metabolism (A5904_2318, A5904_0969, A5904_2211, and A5904_0041) were differentially expressed in the *rpoF* deletion strain (Supplementary Table S4). It was shown that A5904_0041 is a *pelD* homologous gene that is associated with biofilm structure and formation processes in *A. thiooxidans* (Diaz et al., 2018). Therefore, the differential expression of genes involved in biofilm formation and biofilm regulation in *A. caldus* Δ*rpoF* strain, suggests that the absence of RpoF not only influences the flagellum synthesis, but also affects the biofilm formation.

In summary, our experiments focused on the distribution of flagellar gene clusters in *Acidithiobacillus* spp., the regulatory role of *A. caldus* RpoF on flagellar biosynthesis and cell adhesion to surfaces, and the effects of flagella-mediated motility on cell responses to unfavorable environments. Therefore, this study provides an overall knowledge and new insights about the flagella in *Acidithiobacillus* spp., which will promote the study on bacterial environmental adaptation and sulfur metabolism in these acidophilic bacteria.

## Author Contributions

C-LY, RW, J-QL (8th Author), and L-XC designed, conducted, and composed the manuscript. C-LY conducted the experiments and performed the bioinformatics analysis. X-KC, J-QL (4th Author), X-ML, XP, and C-JZ analyzed the data and revised the manuscript.

## Conflict of Interest Statement

The authors declare that the research was conducted in the absence of any commercial or financial relationships that could be construed as a potential conflict of interest.

## Supplementary Material

Figure S1 The phylogenetic tree of representative FlaB proteins which are encoded by *fliC* homologous genes. 35 representative FlaB proteins were used for phylogenetic tree construction with reference sequences. These proteins were labeled with their GenBank accession numbers and bacterial species. ClustalW was used to align these sequences and MEGA 7 was used to build the tree.

Figure S2 Growth rates of the Δ*rpoF* and wildtype strain of *A. caldus* MTH-04 under different growth conditions. Each point in the curve was the first derivative of the corresponding points from the growth curve in Figure 5. GraphPad Prism 7.0 was used for statistical analysis. Growth rate curve for cells in the following conditions: Starkey-K_2_S_4_O_6_ medium containing 1 mM K_2_S_4_O_6_ at 150 rpm **(A)** and 0 rpm **(B)**, Starkey-S^0^ medium with the addition of 0.4 g S^0^ at 150 rpm **(C)** and 0 rpm **(D)**, 1.2 g S^0^ at 150 rpm **(E)**, and 0 rpm **(F)**.

Figure S3 The pH curves of Δ*rpoF* and wildtype strain of *A. caldus* under different growth conditions. The pH changes of Starkey-S^0^ medium containing 1.2 g S^0^ at 150 rpm **(A)** and 0 rpm **(B)**, 0.4 g S^0^ at 150 rpm **(C)**, and 0 rpm **(D)**, respectively.

Figure S4 The attachment of *A. caldus rpoF* deletion and wildtype strain on the S^0^ coupons. Scanning electron micrographs for WT(*rpoF*) **(A)** and Δ*rpoF*(215) **(B)** on S^0^ coupons with 150 rpm.

Figure S5 Consistency check of DEGs between RNA-seq and RT-qPCR. **(A)** RT-qPCR validation for 41 genes. RT-qPCR data are the mean of the results from the three biological replicates. **(B)** agreement comparison between the RNA-seq and RT-qPCR results indicated the data for 39 of the 41 genes lie within the 95% confidence limit.

Figure S6 Flagellar gene clusters in different bacterial species. GenBank accession number for these genomes and the gene IDs for corresponding flagellar cluster are: *Thiomonas intermedia* ATCC 15466 (NZ_CP020046, BVH73_RS00605-RS00370, BVH73_ RS00740-RS00670); *Thiomonas delicata* DSM 16361 (NZ_LT592170, THIARS_RS14765-RS15050, THIARS_RS14615-RS14695); *Leptospirillum ferrooxidans* C2-3 (NC_017094, LFE_RS10820-RS10725, LFE_RS01330-RS01470); *Leptospirillum. ferriphilum* ML-04 (NC_018649, LFML04_RS11235-RS12960, LFML04_RS01075-RS01220); *Vibrio cholerae* O1 (NC_002505, VC0892-0893, VC1008, VC2058-2069, VC2120-2140, VC2187-2205, VC2601); *Escherichia coli* str. K-12 substr. MG1655 (NC_000913, b1066-1083, b1892-1877, b1920-1950). The annotation of each gene is listed in Supplementary Table S2. Different colors mean different structural component of flagellum and other related function, filament is in dark gray, hook is in aquamarine, external basal body (L- and P-ring) is in orange, inner basal body (MS- and C-ring) is in yellow, green represents export apparatus, teal represents regulators and chaperones and white stands for chemotaxis and other unknown function proteins.

Table S1 All primers used in the experiment.

Table S2 Annotations of flagellar genes for all related strains.

Table S3 The raw data of DEGs.

Table S4 Taxonomic traits of *Acidithiobacillus* spp., *Thiobacillus* spp., and *Thiomonas* spp.

Table S5 Gene classification of RNA-seq.
