# Supplementary figures and images for: Essential Role of σ Factor RpoF in Flagellar Biosynthesis and Flagella-Mediated Motility of Acidithiobacillus caldus
Source: Front Microbiol. 2019 May 24;10:1130. doi: 10.3389/fmicb.2019.01130 (PMC6543871; doi:10.3389/fmicb.2019.01130)

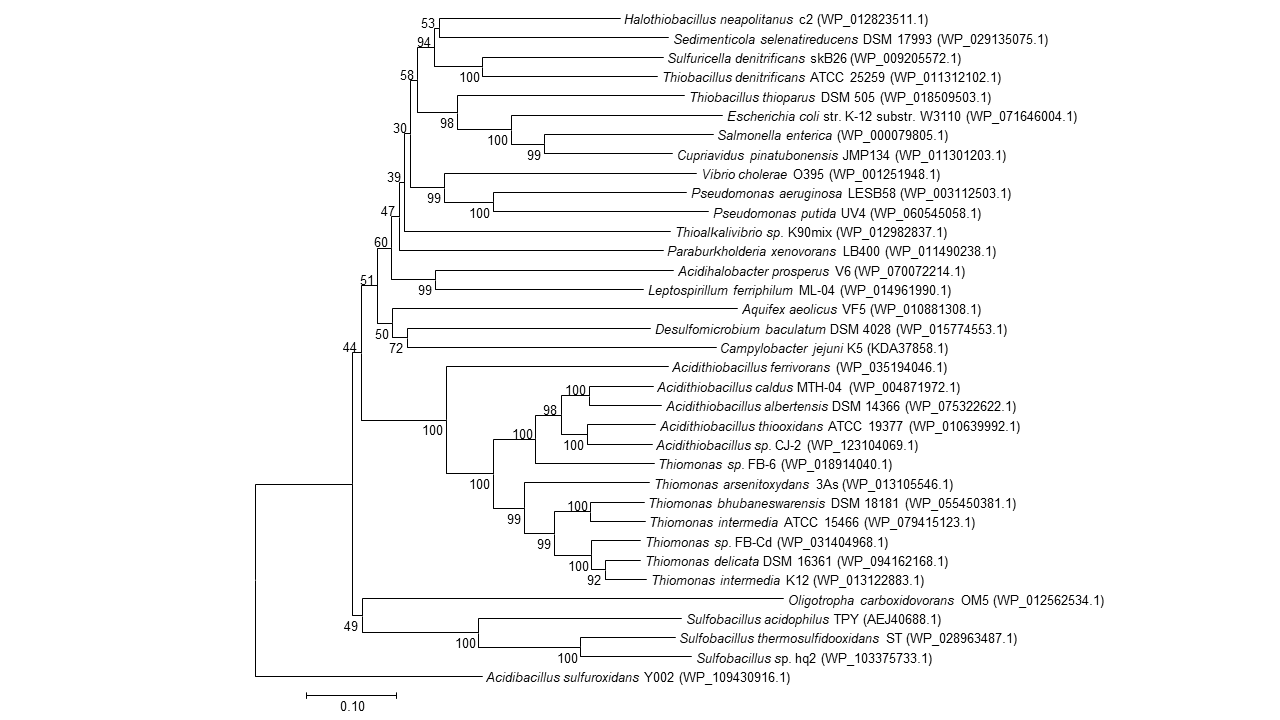

Supplement: Figure S1 — The phylogenetic tree of representative FlaB proteins which are encoded by fliC homologous genes. 35 representative FlaB proteins were used for phylogenetic tree construction with reference sequences. These proteins were labeled with their GenBank accession numbers and bacterial species. ClustalW was used to align these sequences and MEGA 7 was used to build the tree. [file Image_1.TIF]

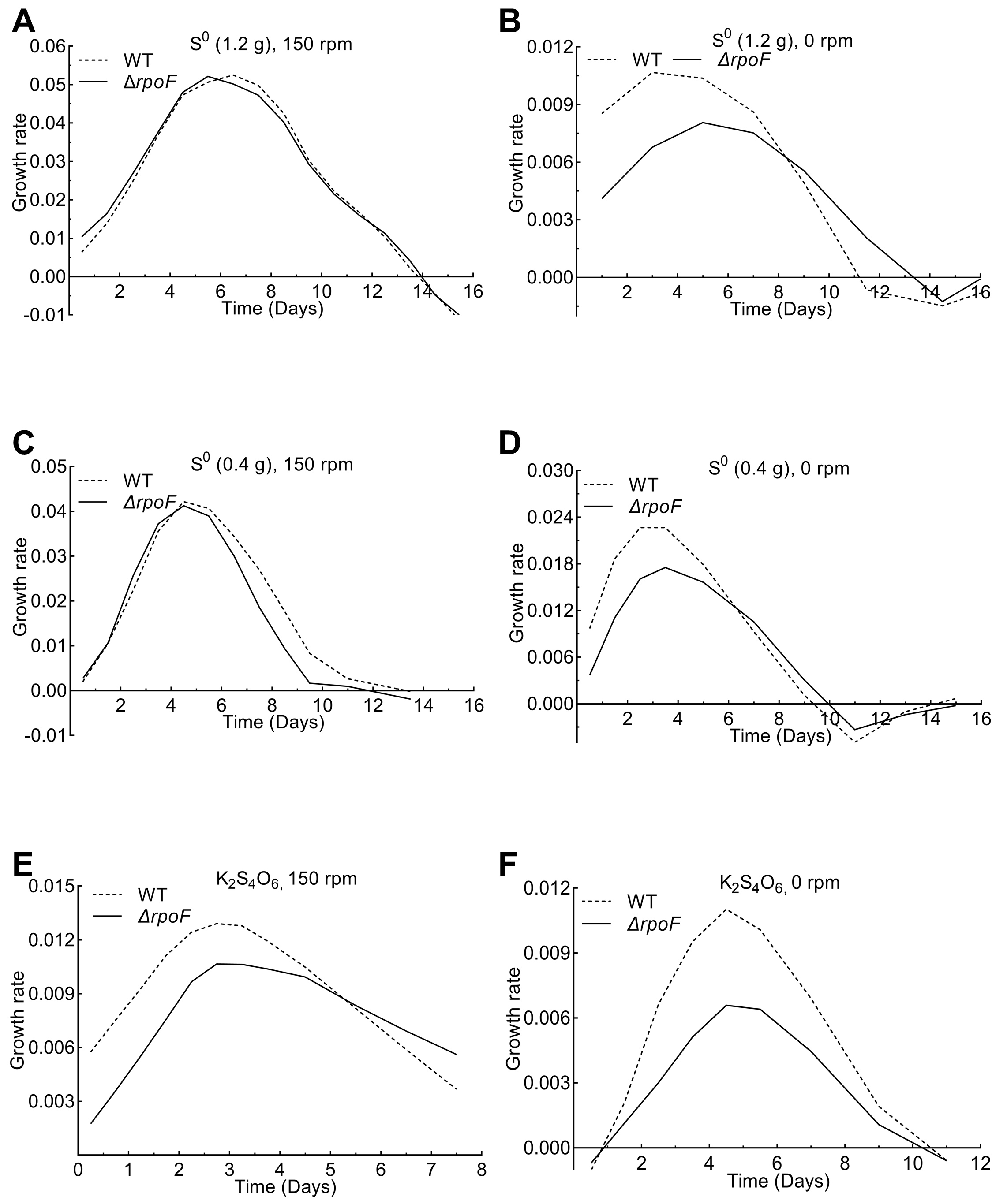

Supplement: Figure S2 — Growth rates of the ΔrpoF and wildtype strain of A. caldus MTH-04 under different growth conditions. Each point in the curve was the first derivative of the corresponding points from the growth curve in Figure 5. GraphPad Prism 7.0 was used for statistical analysis. Growth rate curve for cells in the following conditions: Starkey-K2S4O6 medium containing 1 mM K2S4O6 at 150 rpm (A) and 0 rpm (B), Starkey-S0 medium with the addition of 0.4 g S0 at 150 rpm (C) and 0 rpm (D), 1.2 g S0 at 150 rpm (E), and 0 rpm (F). [file Image_2.TIF]

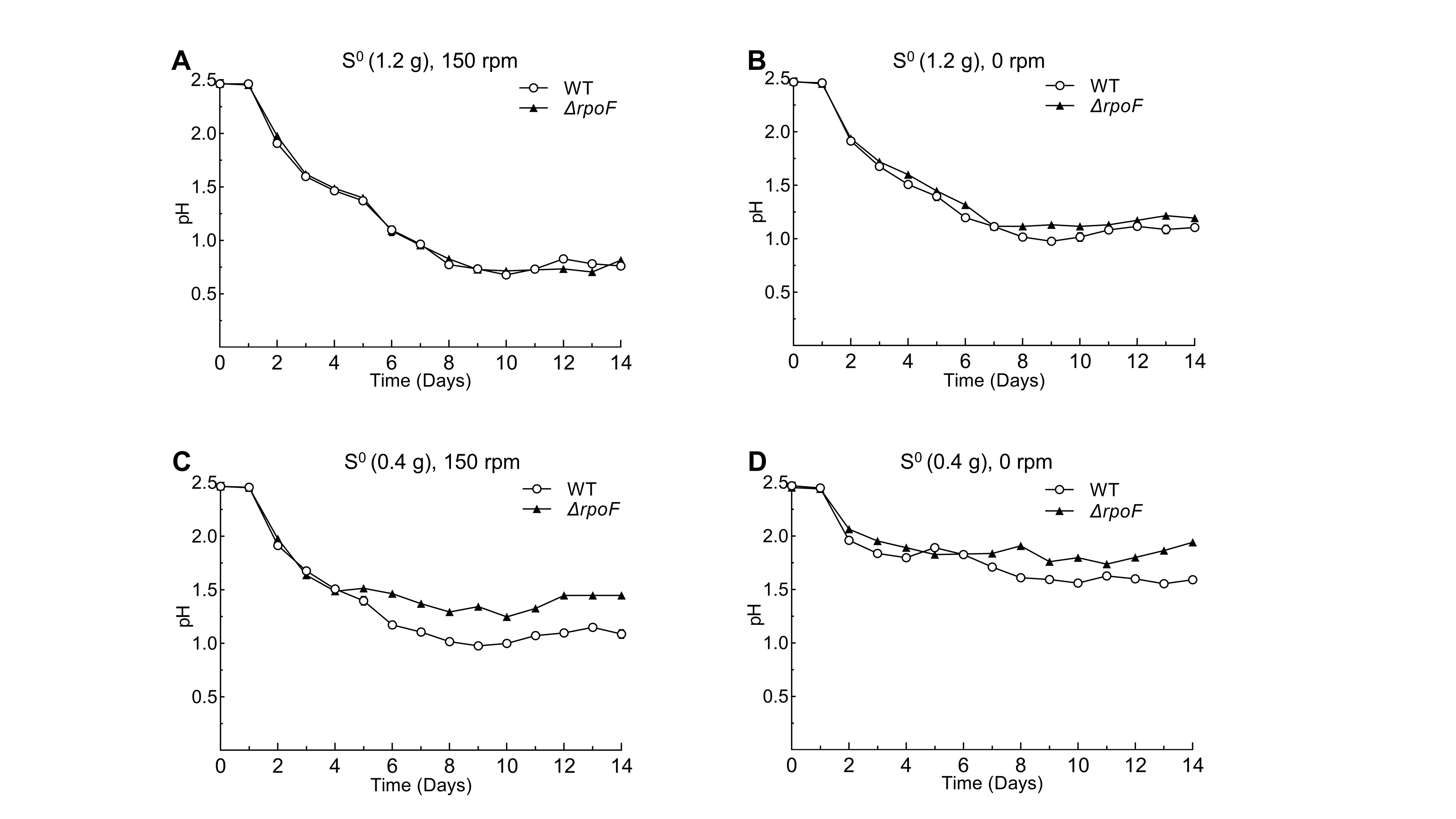

Supplement: Figure S3 — The pH curves of ΔrpoF and wildtype strain of A. caldus under different growth conditions. The pH changes of Starkey-S0 medium containing 1.2 g S0 at 150 rpm (A) and 0 rpm (B), 0.4 g S0 at 150 rpm (C), and 0 rpm (D), respectively. [file Image_3.TIF]

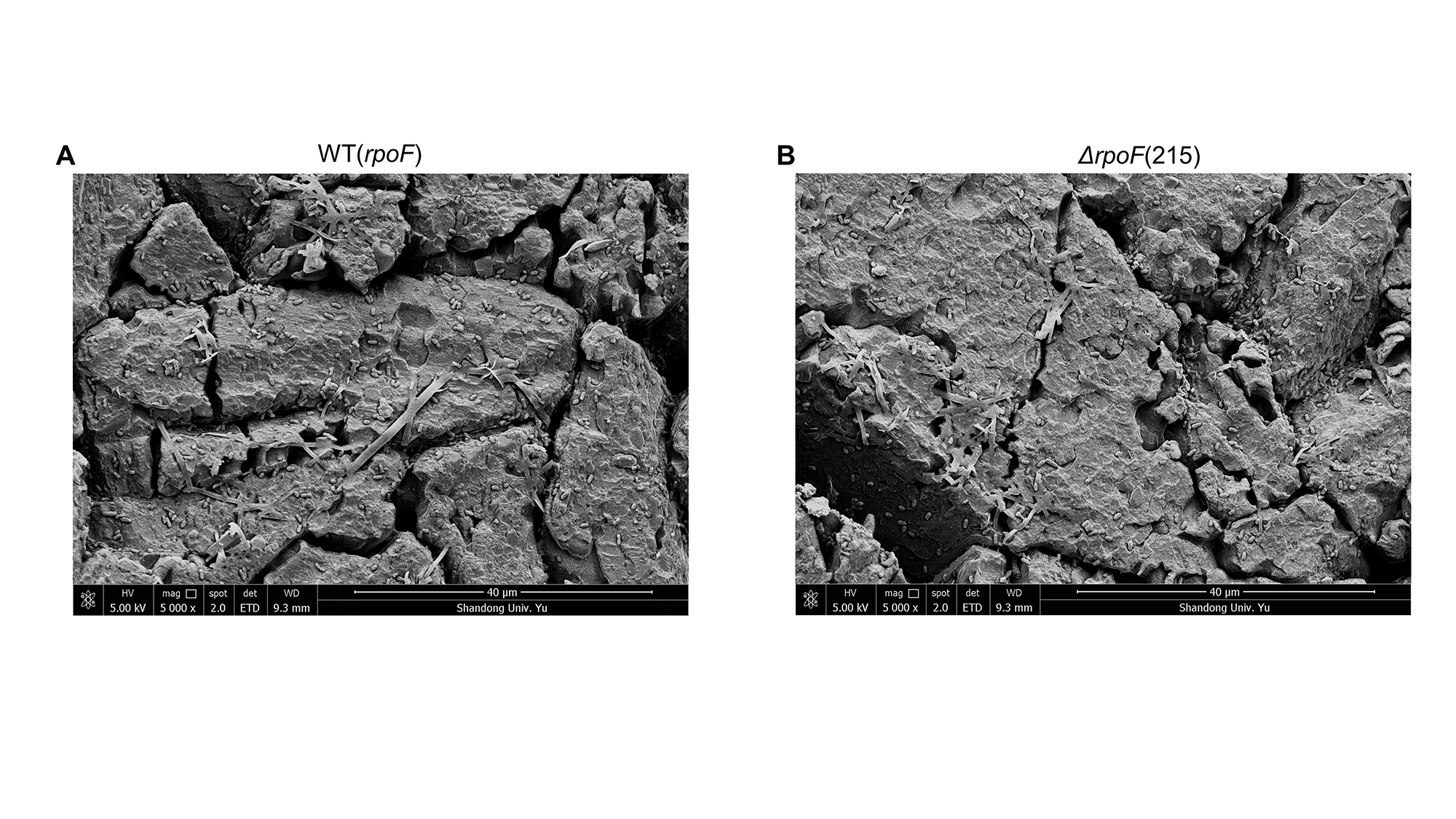

Supplement: Figure S4 — The attachment of A. caldus rpoF deletion and wildtype strain on the S0 coupons. Scanning electron micrographs for WT(rpoF) (A) and ΔrpoF(215) (B) on S0 coupons with 150 rpm. [file Image_4.TIF]

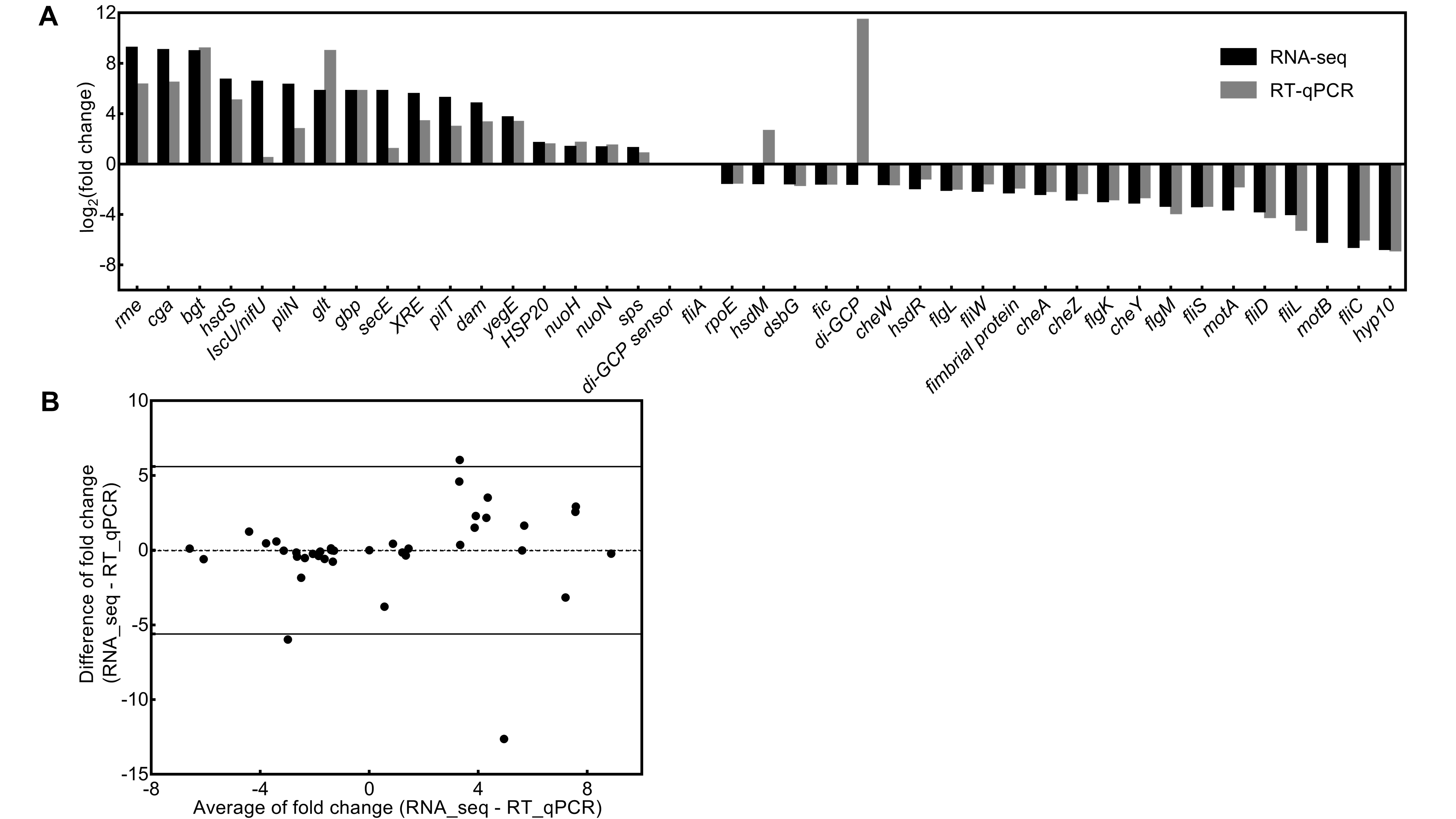

Supplement: Figure S5 — Consistency check of DEGs between RNA-seq and RT-qPCR. (A) RT-qPCR validation for 41 genes. RT-qPCR data are the mean of the results from the three biological replicates. (B) agreement comparison between the RNA-seq and RT-qPCR results indicated the data for 39 of the 41 genes lie within the 95% confidence limit. [file Image_5.TIF]

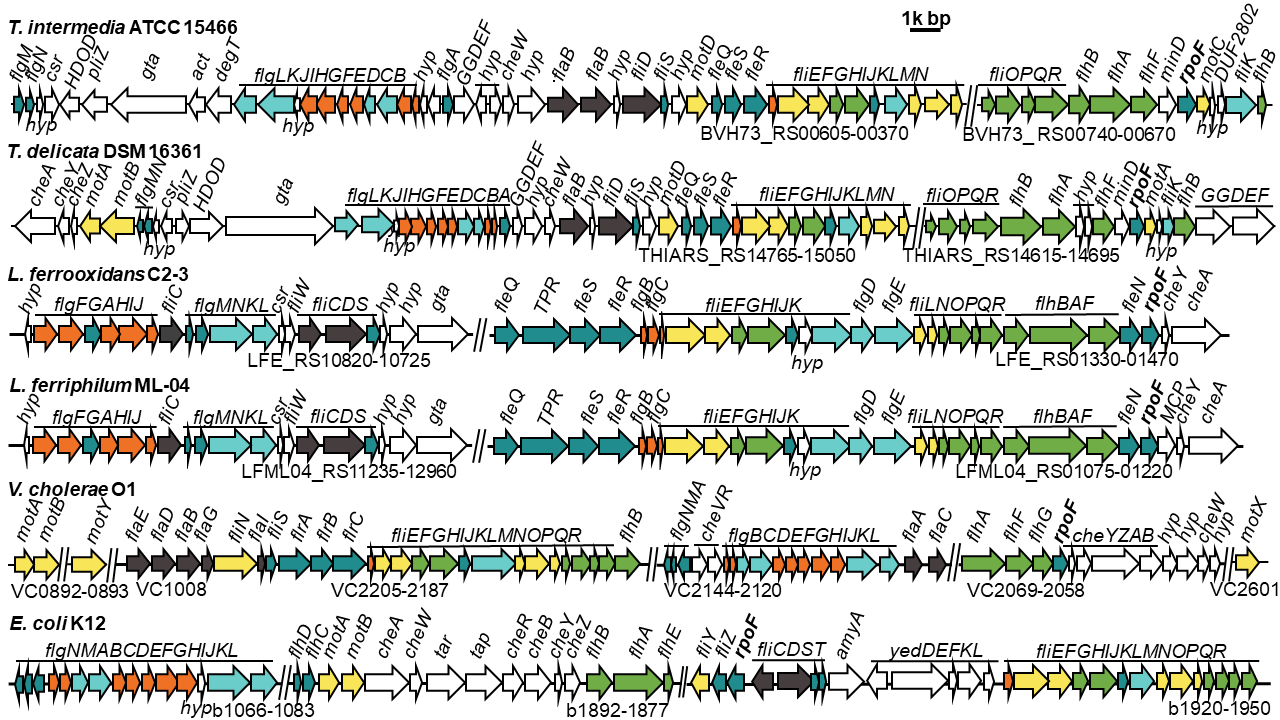

Supplement: Figure S6 — Flagellar gene clusters in different bacterial species. GenBank accession number for these genomes and the gene IDs for corresponding flagellar cluster are: Thiomonas intermedia ATCC 15466 (NZ_CP020046, BVH73_RS00605-RS00370, BVH73_ RS00740-RS00670); Thiomonas delicata DSM 16361 (NZ_LT592170, THIARS_RS14765-RS15050, THIARS_RS14615-RS14695); Leptospirillum ferrooxidans C2-3 (NC_017094, LFE_RS10820-RS10725, LFE_RS01330-RS01470); Leptospirillum. ferriphilum ML-04 (NC_018649, LFML04_RS11235-RS12960, LFML04_RS01075-RS01220); Vibrio cholerae O1 (NC_002505, VC0892-0893, VC1008, VC2058-2069, VC2120-2140, VC2187-2205, VC2601); Escherichia coli str. K-12 substr. MG1655 (NC_000913, b1066-1083, b1892-1877, b1920-1950). The annotation of each gene is listed in Supplementary Table S2. Different colors mean different structural component of flagellum and other related function, filament is in dark gray, hook is in aquamarine, external basal body (L- and P-ring) is in orange, inner basal body (MS- and C-ring) is in yellow, green represents export apparatus, teal represents regulators and chaperones and white stands for chemotaxis and other unknown function proteins. [file Image_6.TIF]
