# Supplementary material for: Essential Role of σ Factor RpoF in Flagellar Biosynthesis and Flagella-Mediated Motility of Acidithiobacillus caldus
Source: Front Microbiol. 2019 May 24;10:1130. doi: 10.3389/fmicb.2019.01130 (PMC6543871; doi:10.3389/fmicb.2019.01130)
Supplement: Table S1 — All primers used in the experiment. [file Table_1.DOCX]

**Table S1 Primer sequence**

| **Primer name** | **Primer sequence (5'-3')** | |  |  |
| --- | --- | --- | --- | --- |
| **RT-qPCR primer** |  |  |  |  |
| A5904_0043 (*fim*)-F | CTGATGATTGTCATCGCCATC | |  |  |
| A5904_0043 (*fim*)-R | GTGGTGTAGACATTACTGAC | |  |  |
| A5904_0148 (*rpoE*)-F | GATGAGGACGATGTGGATGC | |  |  |
| A5904_0148 (*rpoE*)-R | GTTGCCTTCCTGAATGAGGTC | |  |  |
| A5904_0244 (*hsdM*)-F | GTCATCCTTCCCTTCACCGT | |  |  |
| A5904_0244 (*hsdM*)-R | AACAGATTGAAGAAGGGCTG | |  |  |
| A5904_0407 (*dsbG*)-F | GACCACATCGAACACTCCTTC | |  |  |
| A5904_0407 (*dsbG*)-R | CACCAGATGCAGTTCGGATC | |  |  |
| A5904_0765 (*nuoH*)-F | GATGCCCTCAAACTCCTGAG | |  |  |
| A5904_0765 (*nuoH*)-R | CAAACTTGTTGCCCGAGGC | |  |  |
| A5904_0771 (*nuoN*)-F | TCTATTGCTGCTCTATGCAG | |  |  |
| A5904_0771 (*nuoN*)-R | TGCCTATCACCATGCTGATG | |  |  |
| A5904_0969 (di-cGMPase)-F | CCCTCTATCTCAGCAAAGCC | |  |  |
| A5904_0969 (di-cGMPase)-R | CGAGAATGGGCTGGTAGTGT | |  |  |
| A5904_1445 (*cheW*)-F | CGTGCGTCTGGAATGGTC | |  |  |
| A5904_1445 (*cheW*)-R | CCTGCCGAAGAATCTGCTC | |  |  |
| A5904_1448 (*cheA*)-F | CGGGTGGACAGCGATAAC | |  |  |
| A5904_1448 (*cheA*)-R | TCGGAAACCAGCAGGAGT | |  |  |
| A5904_1449 (*cheZ*)-F | ATCAACCTCTGCGGGAAGC | |  |  |
| A5904_1449 (*cheZ*)-R | CTCGTCATAGGCGGAATCTG | |  |  |
| A5904_1450 (*cheY*)-F | CCTGGTGGTGGACGATTTCT | |  |  |
| A5904_1450 (*cheY*)-R | CCTGCATATTCGGCATGTTC | |  |  |
| A5904_1452 (*motD*)-F | TCCTACGCCGACTTCATTAC | |  |  |
| A5904_1452 (*motD*)-R | ACCAGCGTCTCGGATAGC | |  |  |
| A5904_1454 (*fliW*)-F | GCCTGACCTACGACAGAAAAC | |  |  |
| A5904_1454 (*fliW*)-R | ATCTGGTGGAAACTGTGCC | |  |  |
| A5904_1457 (*flgM*)-F | CAACACGCCGATACAGGAC | |  |  |
| A5904_1457 (*flgM*)-R | GACGCAGACCTTTGGCTATC | |  |  |
| A5904_1460 (*flgC*)-F | CGCCACCAGTTCCGATG | |  |  |
| A5904_1460 (*flgC*)-R | TCAACGACGCCAGCCAC | |  |  |
| A5904_1461 (*flgD*)-F | GTGCCAGCACCTTCTACA | |  |  |
| A5904_1461 (*flgD*)-R | GATTTGCCGATGAGCGAG | |  |  |
| A5904_1462 (*flgE*)-F | TGGGAATCCAGGCACAG | |  |  |
| A5904_1462 (*flgE*)-R | AGCGTGATTCCCGATGAC | |  |  |
| A5904_1471 (*flgL*)-F | GTTCCCAGGCAGGCAATG | |  |  |
| A5904_1471 (flgL)-R | GTCCCGACATTACCGCTACTA | |  |  |
| A5904_1473 (*fliC*)-F | CAACCAGGCGGCGATAAC | |  |  |
| A5904_1473 (*fliC*)-R | CGATGTTTTGCCCCTGACTC | |  |  |
| A5904_1475 (*fliD*)-F | TCGCCAGTGCCAGTAACA | |  |  |
| A5904_1475 (*fliD*)-R | GTCAGGGTGAGGCGATAG | |  |  |
| A5904_1476 (*fliS*)-F | CGTGCTGCTGGGATTGGA | |  |  |
| A5904_1476 (*fliS*)-R | CCACGCTGGCAAGGAGAT | |  |  |
| A5904_1487 (*fliK*)-F | GCTGCTCCGACCTTTGAC | |  |  |
| A5904_1487 (*fliK*)-R | CGGTTCTCTGGTTTTTTCTC | |  |  |
| A5904_1499 (*motA*)-F | AGTGACCCGCAACCCATC | |  |  |
| A5904_1499 (*motA*)-R | GCCATCCACGAGCATCTG | |  |  |
| A5904_1536 (*fliL*)-F | AGCAGCGATGGCAGCACC | |  |  |
| A5904_1536 (*fliL*)-R | CCGTTCACCCGTTTTTTG | |  |  |
| A5904_1537 (*hyp10*)-F | CGGGCAGCACTGACCTAC | |  |  |
| A5904_1537 (*hyp10*)-R | CCGAGCACACCGTAATCA | |  |  |
| A5904_1605 (*dam*)-F | CTACGACGAGTGGAGTAATC | |  |  |
| A5904_1605 (*dam*)-R | CGACCTTGTTTTGCATGGAC | |  |  |
| A5904_1705 (*hsdS*)-F | CTGCTTATCCTGGAGACCTC | |  |  |
| A5904_1705 (*hsdS*)-R | GCTTATCTGCCCTCGGTGT | |  |  |
| A5904_1848 (*metS*)-F | GCAACGGACGTGAAAGATCG | |  |  |
| A5904_1848 (*metS*)-R | ATGGTGTGCAAATCCGCCAC | |  |  |
| A5904_1857 (*pliN*)-F | TTGGGTGCGAACATCCTGA | |  |  |
| A5904_1857 (*pliN*)-R | GCATGACCAGTATCACGAAC | |  |  |
| A5904_1860 (*secE*)-F | GTGAGACAGAAGATCAGCATG | |  |  |
| A5904_1860 (*secE*)-R | CTCCAGACGGTACAGAAGTC | |  |  |
| A5904_1863 (*pilT*)-F | GATAATCGTATGACGGCCAAC | |  |  |
| A5904_1863 (*pilT*)-R | TGTCCGAGTTGATAGATGC | |  |  |
| A5904_1867 (*bgt*)-F | CATGCTGGCCGGACCAAGTG | |  |  |
| A5904_1867 (*bgt*)-R | GTGCTGATCACCAGCCAGATG | |  |  |
| A5904_1872 (*glt*)-F | ATGGAGCGGATCGCCGAATC | |  |  |
| A5904_1872 (*glt*)-R | ATGTCGGATCTTCCCGATGCAG | |  |  |
| A5904_2063 (*hsp20*)-F | CAACCAGGTCTACATCAGTG | |  |  |
| A5904_2063 (*hsp20*)-R | TCCACCGGAAGTTGAATGGTG | |  |  |
| A5904_2217 (*hsdR*)-F | CTGGAGCACAACAAGGCCCT | |  |  |
| A5904_2217 (*hsdR*)-R | GAACCGTCCCCTGCTGATAG | |  |  |
| A5904_2254 (*dsbG*)-F | GTGGTTCCTGTGGGCTTCC | | | |
| A5904_2254 (*dsbG*)-R | TCACTTCCGCCAGCACC | |  |  |
| A5904_2557 (*gbp*)-F | TCGATTGGTCGGCTTTCCAC | |  |  |
| A5904_2557 (*gbp*)-R | GACAAAACGAGCACCCGATG | |  |  |
| A5904_2662 (*motB*)-F | GAATCCAGGTGGTAGACAGC | |  |  |
| A5904_2662 (*motB*)-R | GAATCCAGGTGGTAGACAGC | | | |
| A5904_2806 (*XRE*)-F | GAACCTGACCATCAAACAGG | |  |  |
| A5904_2806 (*XRE*)-R | AGGTCGTAGGCCCACAGTAG | |  |  |
| A5904_2810 (*iscU*)-F | GCCCGCTTCAAGACCTACG | |  |  |
| A5904_2810 (*iscU*)-R | CCTCGGCAATCTGACTGTTC | |  |  |
| A5904_2879 (*sps*)-F | CATCGAGGCTGAGGAAGAG | |  |  |
| A5904_2879 (*sps*)-R | CGTTGGCGATTCTCGTAGAG | |  |  |
| A5904_2930 (*cga*)-F | GGAACCACGGCATGAGCAAC | |  |  |
| A5904_2930 (*cga*)-R | GTAGCCAGCCGACATCCGTTG | |  |  |
| **Construction primer** | | | |  |
| rpoF-U-F-XbaⅠ | | CTAGTCTAGACTTGCCGTTGGCGTATTTC | |  |
| rpoF-U-R-KpnⅠ | | CGGGGTACCGCTGCGCTATCATGCACG | |  |
| rpoF-D-F-KpnⅠ | | CGGGGTACCGTCGCTGGGGTAATGGATAT | |  |
| rpoF-D-R-NheⅠ | | AGCTTTGCTAGCCCCGTGACAAGGACATCGTT | |  |
| rpoF-F | | CCCCTAGACAACGTATTAGCGATTCGGAG  ATTATATATCATGATAGCGCAGCGTGCCTG | |  |
| rpoF-R-HindⅢ | | CCCAAGCTTTTACCCCAGCGACCCTA | |  |
| PtetH-F-KpnⅠ | | CGGGGTACCGGGAATCGGGTTGATGAT | |  |
| PtetH-R | | GATATATAATCTCCGAATCG | |  |
| **Confirmation primer** | | | |  |
| P1-F | | GCTTTTCACCACCGATACCTA | |  |
| P1-R | | TGGAACCTCTACCTACGACG | |  |
| P2-F | | GGCTCATTGAAGCAGTCAGTAG | |  |
| P2-R | | GGACCCGACACCGTAGATG | |  |
| P3-F | | TCGGTCTTCTGGATGTTCTCG | |  |
| P3-R | | AATCGCAGGACGGCTTGAC | |  |
