# Supplementary material for: Essential Role of σ Factor RpoF in Flagellar Biosynthesis and Flagella-Mediated Motility of Acidithiobacillus caldus
Source: Front Microbiol. 2019 May 24;10:1130. doi: 10.3389/fmicb.2019.01130 (PMC6543871; doi:10.3389/fmicb.2019.01130)
Supplement: Table S4 — Taxonomic traits of Acidithiobacillus spp., Thiobacillus spp., and Thiomonas spp. [file Table_4.DOCX]

| Trait | Motility | Chemotaxis | Flagella | | Sox system | Oxidation of Fe^2+^ | Referencences |
| --- | --- | --- | --- | --- | --- | --- | --- |
| *A. ferrooxidans* | +/− | - | - (ATCC23270, ATCC53993, Hel18, BY0502, CCM4253, IQ-2C, YQH-1, DLC-5, RVS1) | | - | + | Valdés et al., 2008; Zhang et al., 2018 |
| *A. ferrivorans* | + | + | + (CF27, PRJEB5721, YL15); | - (SS3, 21-59-9, PQ33) | SoxⅡ | + | Hallberg et al., 2010; Liljeqvist et al., 2011 |
| *A. ferriphilus* | + | NG | NG | | NR | + | Falagan et al., 2016 |
| *A. ferridurans* | + | - | - (JCM18981= ATCC33020) | | NR | + | Hedrich et al., 2013 |
| *A. thiooxidans* | + | + | + (ATCC19377, A01, Licanantay, DMC, A02, GD1-3, BY02, JYC-17, DXS-W, ZBY, CLST) | | SoxⅠand SoxⅡ | − | Bryant et al., 1983; Valdes et al., 2011 |
| *A. caldus* | + | + | + (ATCC51756, SM-1, MTH-04, DX, ZBY, ZJ, S1) | | SoxⅠand SoxⅡ | − | Hallberg et al., 1994; Wang et al., 2018 |
| *A. albertensis* | + | + | + DSM14366 | | SoxⅠand SoxⅡ | − | Bryant et al., 1983; Castro et al., 2017. |
| *Thermithiobacillus tepidarius* | + | + | + DSM 3134 | | SoxⅡ | NR | Boden et al., 2016; Hudson et al., 2014 |
| *Thiobacillus thioparus* | + | + | + DSM 505 | | SoxⅠand SoxⅡ | - | Hutt et al., 2017; Vlasceanu et al., 1997 |
| *Thiobacillus denitrificans* | + | + | + (ATCC 25259, DSM 12475, RG18) | | SoxⅠand SoxⅡ | + | Beller et al., 2006; Taylor et al., 1971 |
| *Thiomonas intermedia* | + | + | + (K12, ATCC15466) | | SoxⅡ | NR | Londo, 1963; Wentzien et al., 2004 |
| *Thiomonas delicata* | + | + | + DSM 16361 | | SoxⅡ | + | Battaglia-Brunet et al., 2006; Katayama et al., 2006 |

+, positive; -, negative; +/−, the positive or negative result from different reports; NG, no genome sequence; NR not reported.

Battaglia-Brunet F., Joulian C., Garrido F., Dictor M. C., Morin D., Coupland K., et al. (2006). Oxidation of arsenite by Thiomonas strains and characterization of Thiomonas arsenivorans sp. nov. *Antonie van Leeuwenhoek* 89, 99-108, 10.1007/s10482-005-9013-2.

Beller H. R., Chain P. S., Letain T. E., Chakicherla A., Larimer F. W., Richardson P. M., et al. (2006). The genome sequence of the obligately chemolithoautotrophic, facultatively anaerobic bacterium Thiobacillus denitrificans. *J. Bacteriol.* 188, 1473-88, 10.1128/JB.188.4.1473-1488.2006.

Boden R., Hutt L. P., Huntemann M., Clum A., Pillay M., Palaniappan K., et al. (2016). Permanent draft genome of Thermithiobaclillus tepidarius DSM 3134(T), a moderately thermophilic, obligately chemolithoautotrophic member of the Acidithiobacillia. *Stand Genomic Sci* 11, 74, 10.1186/s40793-016-0188-0.

Bryant R. D., Mcgroarty K. M., Costerton J. W., and Laishle E. J. (1983). Isolation and characterization of a new acidophilic Thiobacillus species (T. albertis). *Can. J. Microbiol.* 29, 1159-1170, 10.1139/m83-178.

Falagan C., and Johnson D. B. (2016). Acidithiobacillus ferriphilus sp. nov., a facultatively anaerobic iron- and sulfur-metabolizing extreme acidophile. *Int. J. Syst. Evol. Microbiol.* 66, 206-11, 10.1099/ijsem.0.000698.

Hallberg K. B., Gonzalez-Toril E., and Johnson D. B. (2010). Acidithiobacillus ferrivorans, sp. nov.; facultatively anaerobic, psychrotolerant iron-, and sulfur-oxidizing acidophiles isolated from metal mine-impacted environments. *Extremophiles : life under extreme conditions* 14, 9-19, 10.1007/s00792-009-0282-y.

Hallberg K. B., and Lindstrom E. B. (1994). Characterization of Thiobacillus caldus sp. nov., a moderately thermophilic acidophile. *Microbiology* 140 ( Pt 12), 3451-3456, 10.1099/13500872-140-12-3451.

Hedrich S., and Johnson D. B. (2013). Acidithiobacillus ferridurans sp. nov., an acidophilic iron-, sulfur- and hydrogen-metabolizing chemolithotrophic gammaproteobacterium. *Int. J. Syst. Evol. Microbiol.* 63, 4018-25, 10.1099/ijs.0.049759-0.

Hudson Corey M., Williams Kelly P., and Kelly Donovan P. (2014). Definitive Assignment by Multigenome Analysis of the Gammaproteobacterial Genus Thermithiobacillus to the Class Acidithiobacillia. *Polish J. Microbiol.* 63, 245–247, issue.

Hutt L. P., Huntemann M., Clum A., Pillay M., Palaniappan K., Varghese N., et al. (2017). Permanent draft genome of Thiobacillus thioparus DSM 505(T), an obligately chemolithoautotrophic member of the Betaproteobacteria. *Stand Genomic Sci* 12, 10, 10.1186/s40793-017-0229-3.

Katayama Y., Uchino Y., Wood A. P., and Kelly D. P. (2006). Confirmation of Thiomonas delicata (formerly Thiobacillus delicatus) as a distinct species of the genus Thiomonas Moreira and Amils 1997 with comments on some species currently assigned to the genus. *Int. J. Syst. Evol. Microbiol.* 56, 2553-7, 10.1099/ijs.0.64299-0.

Liljeqvist M., Valdes J., Holmes D. S., and Dopson M. (2011). Draft genome of the psychrotolerant acidophile Acidithiobacillus ferrivorans SS3. *J. Bacteriol.* 193, 4304-5, 10.1128/JB.05373-11.

Londo Jack (1963). Thiobaeillus intermedius nov. sp. : a novel type of faeultative autotroph. *Archiv fiir Mikrobiologie* 46, 329-337, issue.

Taylor Barrie F., and Hoare Derek S. (1971). Thiobacillus denitrificans as an Obligate Chemolithotroph. *Arch. Mikrobiol.* 80, 262-276, issue.

Valdes J., Ossandon F., Quatrini R., Dopson M., and Holmes D. S. (2011). Draft genome sequence of the extremely acidophilic biomining bacterium Acidithiobacillus thiooxidans ATCC 19377 provides insights into the evolution of the Acidithiobacillus genus. *J. Bacteriol.* 193, 7003-4, 10.1128/JB.06281-11.

Valdés Jorge, Pedroso Inti, Quatrini Raquel, and Holmes David S. (2008). Comparative genome analysis of Acidithiobacillus ferrooxidans, A. thiooxidans and A. caldus: Insights into their metabolism and ecophysiology. *Hydrometallurgy* 94, 180-184, 10.1016/j.hydromet.2008.05.039.

Vlasceanu Luminita, Popa Radu, and Kinkle Brian K. (1997). Characterization of Thiobacillus thioparus LV43 and Its Distribution in a Chemoautotrophically Based Groundwater Ecosystem. Appl. Environ. Microbiol. 63, 3123–3127, issue.

Wang R., Lin J. Q., Liu X. M., Pang X., Zhang C. J., Yang C. L., et al. (2018). Sulfur Oxidation in the Acidophilic Autotrophic Acidithiobacillus spp. Frontiers in microbiology 9, 3290, 10.3389/fmicb.2018.03290.

Wentzien S., and Sand W. (2004). Tetrathionate Disproportionation byThiomonas intermedia K12. Engineering in Life Sciences 4, 25-30, 10.1002/elsc.200400007.
