# Supplementary material for: Essential Role of σ Factor RpoF in Flagellar Biosynthesis and Flagella-Mediated Motility of Acidithiobacillus caldus
Source: Front Microbiol. 2019 May 24;10:1130. doi: 10.3389/fmicb.2019.01130 (PMC6543871; doi:10.3389/fmicb.2019.01130)
Supplement: Table S5 — Gene classification of RNA-seq. [file Table_5.DOCX]

| Gene ID | Gene name | Gene function | FC (RT-qPCR) |
| --- | --- | --- | --- |
| **Motility and chemotaxis** | | | |
| A5904_1445 | *cheW* | chemotaxis protein CheW | 0.38 (0.231 ± 0.026) |
| A5904_1446 |  | flavoprotein | 0.48 |
| A5904_1447 | *hyp* | hypothetical protein | 0.39 |
| A5904_1448 | *cheA* | chemotaxis protein CheA | 0.22 (0.336 ± 0.036) |
| A5904_1449 | *cheZ* | hypothetical protein | 0.16 (0.232 ± 0.012) |
| A5904_1450 | *cheY* | chemotaxis regulator | 0.14 (0.122 ± 0.022) |
| A5904_1452 | *motD* | flagellar motor protein MotD | 0.02 (0) |
| A5904_1454 | *fliW* | hypothetical protein | 0.26 (0.552 ± 0.16) |
| A5904_1455 | *csrA* | hypothetical protein | 0.3 |
| A5904_1456 | *flgN* | hypothetical protein | 0.19 |
| A5904_1457 | *hyp* | hypothetical protein | 0.12 (0.079 ± 0.012) |
| A5904_1469 | *hyp* | hypothetical protein | 0.08 |
| A5904_1470 | *flgK* | flagellar hook protein FlgK | 0.15 |
| A5904_1471 | *flgL* | flagellar hook protein FlgL | 0.28 |
| A5904_1473 | *fliC* | flagellar protein FlaB | 0.01 |
| A5904_1474 | *hyp* | hypothetical protein | 0.05 |
| A5904_1475 | *fliD* | flagellar hook protein FliD | 0.08 (0.062 ± 0.002) |
| A5904_1476 | *fliS* | flagellar export chaperone FliS | 0.11 (0.115 ± 0.029 |
| A5904_1498 | *fliA* | RNA polymerase sigma factor | 0 (0) |
| A5904_1499 | *motA* | flagellar motor protein MotA | 0.09 (0.340 ± 0.052) |
| A5904_1536 | *fliL* | flagellar biosynthesis protein FliL | 0.07 (0.031 ± 0.001) |
| A5904_1537 | *hyp* | hypothetical protein | 0.01 (0.013 ± 0.003) |
| A5904_2662 | *motB* | flagellar motor protein MotB | 0.02 |
| **Energy metabolism** | | | |
| A5904_0714 | *nuoB* | NADH dehydrogenase subunit B | 0.42 |
| A5904_0764 | *nuoG* | NADH-ubiquinone oxidoreductase chain G | 2.04 |
| A5904_0765 | *nuoH* | NADH-ubiquinone oxidoreductase chain H | 2.25 (2.854 ± 0.479) |
| A5904_0766 | *nuoI* | NADH-ubiquinone oxidoreductase chain I | 2.26 |
| A5904_0771 | *nuoN* | NADH-ubiquinone oxidoreductase chain N | 2.2 (2.444 ± 0.201) |
| A5904_1072 | *ccsA/resC* | cytochrome c-type biogenesis protein | 2.17 |
| A5904_1252 | *cydB* | cytochrome d ubiquinol oxidase subunit II | 0.2 |
| A5904_1253 | *cydA* | cytochrome d ubiquinol oxidase subunit I | 0.21 |
| A5904_1799 | *ccdA* | cytochrome c biogenesis protein, transmembrane region | 0.41 |
| A5904_2186 | *cydB* | cytochrome d ubiquinol oxidase subunit II | 0.28 |
| A5904_2209 | *ndhF* | NADH dehydrogenase subunit 5 | 0.23 |
| A5904_2899 | *ahpC* | alkyl hydroperoxide reductase subunit C-like protein | 0.47 |
| **DNA modification** | | | |
| A5904_0005 | *wtpA* | ABC transporter substrate-binding protein | 0.16 |
| A5904_0244 | *hsdM* | restriction endonuclease subunit M | 0.4 (5.449± 2.318) |
| A5904_0983 | ABC-2.LPSE.P | ABC transporter permease | 0.5 |
| A5904_1599 | *parB, spoJ* | hypothetical protein | 52.09 |
| A5904_1643 | *dpo* | uracil-DNA glycosylase, family 4 | 42.14 |
| A5904_1705 | *hsdS* | Type I restriction-modification system, specificity subunit S | 91.3 (29.013 ± 6.302) |
| A5904_1706 | *hsdM* | DNA-methyltransferase subunit M | 325.41 |
| A5904_1848 | *metS* | Type I restriction modification DNA specificity domain | 527.86 (69.638 ± 11.306) |
| A5904_2217 | *hsdR* | type I restriction-modification system endonuclease | 0.3 (0.516 ± 0.142) |
| A5904_2242 | *hyp* | hypothetical protein | 39.21 |
| A5904_2243 | *hyp* | hypothetical protein | 40.38 |
| A5904_2245 | *hyp* | hypothetical protein | 38.63 |
| **Transportion** | | | |
| A5904_2283 | *exbB* | biopolymer transport protein | 0.49 |
| A5904_2356 | *kch, trkA, mthK, pch* | potassium channel protein | 0.35 |
| A5904_2774 | *nrtA, nasF, cynA* | nitrate transporter | 0.41 |
| A5904_2775 | *NRT, narK, nrtP, nasA* | MFS transporter | 0.26 |
| **Transcriptional regulator** | | | |
| A5904_0065 | SIG3.2, *rpoE* | DNA-directed RNA polymerase sigma-70 factor | 0.32 |
| A5904_1429 | SIG3.2, *rpoE* | DNA-directed RNA polymerase sigma-70 factor | 0.4 (0.412 ± 0.0530) |
| A5904_0628 |  | transcriptional regulator, AbrB family | 2.16 |
| A5904_1002 |  | Fis family transcriptional regulator | 0.43 |
| A5904_2210 |  | LysR family transcriptional regulator | 0.21 |
| A5904_2215 |  | transcriptional regulator | 0.36 |
| A5904_2216 |  | RNA helicase | 0.33 |
| A5904_2806 |  | XRE family transcriptional regulator | 41.55 (9.235 ± 2.824) |
| **DNA replication** | | | |
| A5904_0538 |  | recombinase | 62.04 |
| A5904_1590 |  | conserved hypothetical protein | 48.58 |
| A5904_1592 |  | conserved hypothetical protein | 76.08 |
| A5904_1594 | *s87* | Helix-turn-helix motif:Peptidase S24, S26A and S26B | 85.45 |
| A5904_1597 |  | conserved hypothetical protein | 34.06 |
| A5904_1598 |  | hypothetical protein | 54.43 |
| A5904_1599 | *parB, spoJ* | conserved hypothetical protein | 52.09 |
| A5904_1601 |  | hypothetical protein | 33.94 |
| A5904_1602 |  | conserved hypothetical protein | 40.38 |
| A5904_1604 |  | conserved hypothetical protein | 53.26 |
| A5904_1605 | *dam* | D12 class N6 adenine-specific DNA methyltransferase | 24.58 |
| A5904_1606 | *traC* | DNA primase | 231.17 |
| A5904_1607 |  | hypothetical protein | 191.97 |
| A5904_1608 |  | hypothetical protein | 137.31 |
| A5904_1609 |  | hypothetical protein | 71.40 |
| A5904_1611 |  | hypothetical protein | 794.22 |
| A5904_1612 |  | hypothetical protein | 36.28 |
| A5904_1613 |  | conserved hypothetical protein | 50.33 |
| A5904_1619 | *hupB* | putatvie integrase | 100.08 |
| A5904_1620 |  | hypothetical protein | 61.45 |
| A5904_1621 |  | hypothetical protein | 32.19 |
| A5904_1622 |  | hypothetical protein | 22.24 |
| A5904_1623 |  | hypothetical protein | 136.36 |
| A5904_1624 |  | hypothetical protein | 3418.15 |
| A5904_2173 | *recG* | ATP-dependent DNA helicase | 62.04 |
| A5904_2256 |  | conserved hypothetical protein | 36.28 |
| A5904_2258 |  | conserved hypothetical protein | 174.41 |
| A5904_2260 | *recG* | ATP-dependent DNA helicase | 22.24 |
| A5904_2264 | *hyp* | hypothetical protein | 69.06 |
| A5904_2266 | *hyp* | hypothetical protein | 344.72 |
| A5904_2267 | E3.6.1.- | putative DNA helicase | 174.99 |
| A5904_2270 | *dnaN* | DNA polymerase III subunit beta | 26.34 |
| A5904_2553 | *ssb* | single-stranded DNA-binding protein | 228.25 |
| A5904_2554 |  | conserved hypothetical protein | 202.12 |
| A5904_2556 |  | putative phosphatase | 33.94 |
| A5904_2557 |  | GTP-binding protein | 48.58 (48.877 ± 5.289) |
| A5904_2569 |  | DNA primase | 3.46 |
| A5904_2782 |  | response regulator NasT | 0.30 |
| A5904_2783 | *recD* | ATPase AAA | 0.39 |
| A5904_2784 |  | conserved hypothetical protein | 0.30 |
| **Conjugation** | | | |
| A5904_0539 | *hyp* | hypothetical protein | 25.75 |
| A5904_1693 | *trbI, virB1, lvhB1* | conjugative transfer protein | 34.53 |
| A5904_1694 | *trbG, virB9, lvhB9* | conjugal transfer protein | 48.58 |
| A5904_1695 | *trbF, virB5, lvhB5* | conjugative transfer protein | 75.5 |
| A5904_1697 | *traB* | conjugal transfer protein | 49.75 |
| A5904_1699 | *hyp* | hypothetical protein | 21.65 |
| A5904_1701 | *hyp* | hypothetical protein | 39.8 |
| A5904_1702 | *hyp* | hypothetical protein | 26.34 |
| A5904_1707 | *hyp* | hypothetical protein | 149.24 |
| A5904_1885 | *parB, spoJ* | chromosome partitioning protein | 56.18 |
| A5904_1886 | *parA, soj* | chromosome partitioning protein | 21.65 |
| A5904_2170 | *trbF, virB5, lvhB5* | conjugal transfer protein | 2.28 |
| A5904_2271 | *hyp* | hypothetical protein | 29.85 |
| A5904_2272 | *traG, virD4, lvhD4* | conjugal transfer protein | 150.41 |
| A5904_2419 | *hyp* | hypothetical protein | 1037.58 |
| A5904_2420 | *hyp* | hypothetical protein | 156.85 |
| A5904_2913 |  | plasmid conjugation-like protein | 50.92 |
| A5904_2915 |  | hypothetical protein | 74.33 |
| A5904_2916 | *virB4* | conjugal transfer protein | 234.10 |
| A5904_2918 |  | hypothetical protein | 141.04 |
| A5904_2920 | *trbB* | type II secretion system protein E | 57.94 |
| **Pilus and secretion** | | | |
| A5904_0039 | *pliZ* | pilus biosynthesis protein PilZ | 0.48 |
| A5904_0041 | *pulF* | type II secretion system protein F | 0.39 |
| A5904_0042 | *hyp* | hypothetical protein | 0.34 |
| A5904_0043 | *fimT* | fimbrial protein | 0.24 (0.315 ± 0.0526) |
| A5904_1857 | *pliN* | pilus assembly protein | 69.06 (6.021 ± 1.512) |
| A5904_1858 | *hyp* | hypothetical protein | 75.5 |
| A5904_1859 | *hyp* | hypothetical protein | 21.65 |
| A5904_1860 | *secE* | secretion system protein E | 48.58 (2.012 ± 0.173) |
| A5904_1861 | *pulF* | Type II secretion inner membrane protein | 39.21 |
| A5904_1862 | *hyp* | hypothetical protein | 29.85 |
| A5904_1863 | *pilT* | twitching motility protein | 33.36 (6.790 ± 1.452) |
| A5904_1864 | *hyp* | hypothetical protein | 757.97 |
| A5904_1865 | *hyp* | hypothetical protein | 748.52 |
| A5904_2269 | *virB1* | Bores hole in peptidoglycan layer allowing type IV secretion complex assembly to occur (VirB1) | 40.38 |
| A5904_2353 | *virD4* | Type IV secretion system protein | 2.18 |
| A5904_2354 | *virB1* | Lytic transglycosylase catalytic | 5.06 |
| **Responsory** | | | |
| A5904_0969 | di_GCP sensor | response regulator receiver modulated diguanylate cyclase/phosphodiesterase with PAS/PAC sensor(s) | 0 (0) |
| A5904_1834 | tspO | sensory protein TspO | 0.44 |
| A5904_2063 | HSP20 | Small heat shock protein | 2.82 (2.610 ± 0.319) |
| A5904_2211 | *yedQ* | conserved hypothetical protein,Diguanylate-cyclase (DGC) or GGDEF domain | 0.39 (2455.022 ± 183.162) |
| A5904_2318 | GGDEF domian | conserved hypothetical protein,Diguanylate-cyclase (DGC) or GGDEF domain | 19 |
| **Carbon metabolism** | | | |
| A5904_1534 | *aroK* | E2.7.1.71, *aroK, aroL* | 49.16 |
| A5904_1866 |  | glycosyltransferase | 309.01 |
| A5904_1867 |  | bactoprenol glucosyl transferase | 433.92 (507.935 ± 15.236) |
| A5904_1871 |  | hypothetical protein | 431.35 |
| A5904_1872 |  | glycosyl transferase family 2 | 49.16 (441.291 ± 52.316) |
| A5904_2294 |  | dihydroxy-acid dehydratase | 0.48 |
| A5904_2471 |  | glycosyl transferase, group 2 family protein | 0.4 |
| A5904_2879 | E2.4.1.14 | sucrose-phosphate synthase | 2.12 (1.573 ± 0.613) |
| A5904_2930 |  | glucan 1,4-alpha-glucosidase | 460.61 (77.462 ± 10.164) |
| **Nitrogen metabolism** | | | |
| A5904_0100 |  | nitrate/nitrite transporter | 0.34 |
| A5904_2733 |  | regulation of nitrogen compound metabolic process | 0.37 |
| A5904_2777 | *nirB* | nitrite reductase large subunit | 0.2 |
| A5904_2779 | *nasA* | nitrate reductase subunit alpha | 0.36 |
| **Sulfur metabolism** | | | |
| A5904_0407 | *dsbG* | thiol:disulfide interchange protein DsbG | 0.4 (0.362 ± 0.018) |
| A5904_1003 | *hyp* | hypothetical protein | 2.1 |
| A5904_1004 | *rhd* | Rhodanese-like domain protein | 2.44 |
| A5904_1265 | *hyp* | hypothetical protein | 2.07 |
| A5904_2254 | *dsbG* | thiol:disulfide interchange protein | 91.89 |
| A5904_2810 | *iscU, nifU* | iron-sulfur cluster scaffold-like protein | 81.35 (1.228 ± 0.15) |
| **Others** | | | |
| A5904_0003 |  | conserved hypothetical protein | 0.24 |
| A5904_0066 |  | conserved hypothetical protein | 0.30 |
| A5904_0067 |  | conserved hypothetical protein | 0.30 |
| A5904_0071 |  | conserved hypothetical protein | 0.42 |
| A5904_0083 |  | hypothetical protein | 0.13 |
| A5904_0084 |  | hypothetical protein | 0.21 |
| A5904_0107 |  | conserved hypothetical protein | 0.45 |
| A5904_0305 |  | conserved hypothetical protein | 2.08 |
| A5904_0407 |  | hypothetical protein | 0.40 |
| A5904_0537 |  | conserved hypothetical protein | 79.01 |
| A5904_0575 |  | Ferredoxin-dependent glutamate synthase | 0.29 |
| A5904_0576 |  | bifunctional proline dehydrogenase/pyrroline-5-carboxylate dehydrogenase | 0.26 |
| A5904_0708 |  | hypothetical protein | 0.37 |
| A5904_1339 |  | conserved hypothetical protein | 0.45 |
| A5904_1442 |  | conserved hypothetical protein | 0.02 |
| A5904_1573 |  | conserved hypothetical protein | 0.40 |
| A5904_1632 |  | hypothetical protein | 24.58 |
| A5904_1783 |  | conserved hypothetical protein | 0.34 |
| A5904_1785 |  | conserved hypothetical protein | 0.32 |
| A5904_1843 |  | hypothetical protein | 54.43 |
| A5904_1849 |  | conserved hypothetical protein | 143.39 |
| A5904_1850 |  | conserved hypothetical protein | 25.17 |
| A5904_1853 |  | conserved hypothetical protein | 46.82 |
| A5904_1856 |  | hypothetical protein | 23.41 |
| A5904_1877 |  | conserved hypothetical protein | 29.85 |
| A5904_1878 |  | conserved hypothetical protein | 695.35 |
| A5904_1879 |  | conserved hypothetical protein | 197.23 |
| A5904_1881 |  | conserved hypothetical protein | 132.27 |
| A5904_1882 |  | conserved hypothetical protein | 35.11 |
| A5904_1884 |  | hypothetical protein | 28.09 |
| A5904_1887 |  | conserved hypothetical protein | 48.58 |
| A5904_2016 |  | conserved hypothetical protein | 0.33 |
| A5904_2167 |  | hypothetical protein | 2.69 |
| A5904_2189 |  | conserved hypothetical protein | 0.23 |
| A5904_2197 |  | hypothetical protein | 86.62 |
| A5904_2204 |  | conserved hypothetical protein | 0.29 |
| A5904_2208 |  | Uncharacterized protein conserved in bacteria | 0.30 |
| A5904_2218 |  | HrgA protein | 0.39 |
| A5904_2219 |  | conserved hypothetical protein | 0.22 |
| A5904_2232 |  | hypothetical protein | 0.39 |
| A5904_2234 |  | conserved hypothetical protein | 0.38 |
| A5904_2239 |  | hypothetical protein | 0.11 |
| A5904_2468 |  | conserved hypothetical protein | 11.36 |
| A5904_2469 |  | Tetratricopeptide-like helical | 14.63 |
| A5904_2549 |  | conserved hypothetial protein | 343.53 |
| A5904_2550 |  | conserved hypothetical protein | 3010.94 |
| A5904_2552 |  | hypothetical protein | 24.58 |
| A5904_2567 |  | hypothetical protein | 13.00 |
| A5904_2568 |  | hypothetical protein | 24.01 |
| A5904_2781 |  | uroporphyrin-III C-methyltransferase | 0.28 |
| A5904_2805 |  | hypothetical protein | 94.81 |
| A5904_2807 |  | hypothetical protein | 815.82 |
| A5904_2808 |  | hypothetical protein | 70.23 |
| A5904_2927 |  | periplasmic mercuric ion binding protein | 222.98 |
| A5904_2928 | *merA* | mercuric reductase MerA | 320.73 |
| A5904_2929 |  | organomercurial lyase | 179.06 |
| A5904_2948 | *czcA* | heavy metal efflux pump, CzcA family, domain: Heavy metal efflux pump CzcA | 2.21 |

Numbers in brackets from qRT-PCR
